# Traveling waves and effective mass for the regularized Landau-Pekar equations

**DOI:** 10.1007/s00526-024-02735-3

**Published:** 2024-04-26

**Authors:** Simone Rademacher

**Affiliations:** Department of Mathematics, LMU Munich, Theresienstrasse 39, 80333 Munich, Germany

**Keywords:** 35Q40, 35Q55, 35C07, 35A15

## Abstract

We consider the regularized Landau-Pekar equations with positive speed of sound and prove the existence of subsonic traveling waves. We provide a definition of the effective mass for the regularized Landau-Pekar equations based on the energy-velocity expansion of subsonic traveling waves. Moreover we show that this definition of the effective mass agrees with the definition based on an energy-momentum expansion of low energy states.

## Introduction and main results

The polaron is quasi-particle that models an electron moving through an ionic crystal while interacting with its self-induced polarization field. The polarization field can be either described as a quantum field by the Fröhlich model [1] (called quantum polaron) or as a classical field by the Landau-Pekar equations [2–4] (called classical polaron). The Landau-Pekar equations describe the polaron as a pair $(\psi , \varphi ) \in H^1 (\mathbb {R}^3) \times L^2_{\sqrt{\epsilon }} ( \mathbb {R}^3)$ where $\psi$ denotes the $L^2$-normalized wave function of the electron and $\varphi$ the classical field which is for a positive function $\varepsilon >0$ an element of

1.1 $$\begin{aligned} L_{\sqrt{\varepsilon }}^2 ( \mathbb {R}^3 ) := \left\{ \varphi \; \vert \; \Vert \sqrt{\varepsilon } \varphi \Vert _2 < \infty \right\} \; . \end{aligned}$$

The Landau-Pekar equations are given by the coupled system of differential equations

1.2 $$\begin{aligned} i \partial _t \psi _t = {\textrm{h}}_{\sqrt{\alpha } \varphi _t}\psi _t, \quad i \varepsilon ^{-1} \partial _t \varphi _t = \varphi _t + \sqrt{\alpha } \sigma _{\psi _t} \end{aligned}$$

where $\alpha >0$ denotes the coupling constant, $m>0$ the electron’s mass,

1.3 $$\begin{aligned} \sigma _\psi := (2\pi )^{3/2} \frac{v}{ \varepsilon } \widehat{\varrho }_\psi , \quad \textrm{h}_{ \varphi } = - \frac{\Delta }{2m} + V_{\varphi }, \quad \text {with} \quad V_{\varphi } (x) = 2 \mathrm{Re\,}\int e^{ik \cdot x} v(k) \varphi (k) dk \end{aligned}$$

and where $$\varrho _\psi : =\vert \psi \vert ^2$$,

1.4 $$\begin{aligned} \varepsilon = 1, \quad \text {and} \quad v(k) = \frac{1}{\vert k \vert } \; . \end{aligned}$$

The strong coupling limit is linked with a classical field approximation: For $\alpha \rightarrow \infty$, the classical Landau-Pekar equations can be derived from the quantum dynamics generated by the Fröhlich model [5–10].

### Effective mass problem for the Landau-Pekar equations

The dynamics of the polaron is closely related to the outstanding problem of its effective mass: Due to the interaction with the self-induced polarization field, the electron slows down. In physics this phenomenon is described by the emergence of a quasi-particle, the polaron, with an increased effective mass $m_\textrm{eff}>0$.

Based on the classical polaron, Landau and Pekar [2–4] formulated a famous quantitative prediction for the effective mass in the strong coupling limit. Their heuristic ideas (described in more detail in [11]) rely on the existence of traveling waves of the Landau-Pekar equations, i.e. solutions of (1.2) with initial data $(\psi _\textrm{v}, \varphi _\textrm{v}) \in H^1( \mathbb {R}^3) \times L^2_{\sqrt{\varepsilon }} ( \mathbb {R}^3)$, $\Vert \psi _\textrm{v}\Vert _2 =1$ satisfying

1.5 $$\begin{aligned} \left( \psi _t (x), \; \varphi _t (k)\right) = \left( e^{i e_\textrm{v}t } \psi _\textrm{v}(x -\textrm{v}t ) , \; e^{i \textrm{v}\cdot k t }\varphi _\textrm{v}(k ) \right) \; \end{aligned}$$

with phase $e_\textrm{v}\in \mathbb {R}$ and velocity $\textrm{v}\in \mathbb {R}^3$. Traveling waves were, however, conjectured to not exists for $\textrm{v}\not = 0$ for the Landau-Pekar equations [11] due to a vanishing speed of sound.

Related to that, the corresponding energy functional to (1.2) does not dominate the total momentum. Thus a computation of the energy as function of conserved total momentum yields a constant function and therefore an infinite mass. Contrarily for the quantum Fröhlich model such an energy-momentum expansion allows to approach the quantum polaron’s effective mass (see [12–14] resp. [15–17] for recent progress based on different techniques).

However for the classical polaron, i.e. the Landau-Pekar equations, neither traveling wave solutions nor an energy-momentum expansion serve for a mathematical rigorous definition of the effective mass. To overcome these problems [11] provides a definition of the effective mass that is based on a novel energy-velocity expansion and verifies the quantitative prediction by Landau and Pekar for the classical polaron.

The goal of this paper is to verify Landau and Pekar’s heuristic approach for the effective mass, originally formulated for the non-regularized classical polaron, mathematical rigorously for a regularized classical polaron model, namely the regularized Landau-Pekar equations.

More precisely, we choose instead of (1.4) the functions $\varepsilon ,v$ to be sufficiently regular (see Assumptions 1.1, 1.2 below) and show that subsonic traveling wave solutions with non-vanishing velocity $\textrm{v}\not = 0$ do exist (Theorem 1) and serve for a definition of the effective mass (Theorem 2). Moreover, the resulting formula agrees with a definition of the effective mass through an energy-momentum expansion (Theorem 3) and, furthermore, with results obtained for the quantum regularized Fröhlich model [18].

### Regularized Landau-Pekar equations

The regularized Landau-Pekar equations describe more generally a particle moving through an excitable medium. We impose the following assumptions on the functions $\varepsilon ,v$ and

1.6 $$\begin{aligned} g= ( 2 \pi )^{3/2} \widehat{\left( v / \varepsilon ^{-1/2} \right) } \; . \end{aligned}$$

#### Assumption 1.1

*(Regularity)* Let $\varepsilon , v$ be radial with $\varepsilon >0$ and such that $g\in H^2 (\mathbb {R}^3)$ and $\vert \widehat{g} (k) \vert \ge (1+ \vert k \vert )^{-9/2}$ for all $k \in \mathbb {R}^3$.

Furthermore we consider underlying media of positive critical velocity $\textrm{v}_\textrm{crit}>0$ formulated in the assumption below. The critical velocity is often referred to as speed of sound of the medium.

#### Assumption 1.2

Let $\varepsilon >0$ satisfy $\inf _{k \in \mathbb {R}^3} \frac{\varepsilon (k)}{\vert k \vert }:= \textrm{v}_\textrm{crit}$ for a constant $\textrm{v}_\textrm{crit}>0$.

We remark that Assumptions 1.1 and 1.2 exclude the (non-regularized) Landau-Pekar equations with $\varepsilon ,v$ given by (1.4) that, in particular, have vanishing speed of sound with the above definition.

The dynamical equations (1.2) for $\varepsilon ,v$ satisfying Assumption 1.1, 1.2 are well defined (see Lemma 2.1 below). Moreover the energy functional

1.7 $$\begin{aligned} \mathcal {G}_\alpha ( \psi , \varphi ) = \langle \psi |\textrm{h}_{\sqrt{\alpha } \varphi }| \psi \rangle + \Vert \varepsilon ^{1/2}\varphi \Vert _2^2 \quad \text {with} \quad \Vert \psi \Vert _2=1 \end{aligned}$$

where $h_{\varphi }$ is given by (1.3) is preserved along the dynamics. For the regularized Landau-Pekar equations we show that there exists subsonic traveling wave solutions with $0< \vert \textrm{v}\vert < \textrm{v}_\textrm{crit}$.

#### Theorem 1

Let $\varepsilon , v$ satisfy Assumptions 1.1 and 1.2 and $\vert \textrm{v}\vert < \textrm{v}_\textrm{crit}$. Then there exists a traveling wave solution of the form (1.5) with $\textrm{v}\not = 0$.

Theorem 1 follows from Proposition 2.3 and is proven in Sect. 4.

We can not treat the case of supersonic traveling waves $\vert \textrm{v}\vert > \textrm{v}_\textrm{crit}$. However we conjecture that supersonic traveling waves do not exist. This conjecture is based on the observation that for $\vert \textrm{v}\vert > \textrm{v}_\textrm{crit}$, the energy functional does not dominate the total momentum, similarly as for the non-regularized model discussed before.

#### Effective mass problem for the regularized Landau-Pekar equations

We provide two definitions for the effective mass. The first definition (Theorem 2) is based on an energy-velocity expansion of traveling wave solutions and inspired by ideas of Landau and Pekar. The second (Theorem 3) is based on an energy-momentum expansion for low energy states. Both definitions lead to the same formula for the effective mass and, in particular, verify the physicists’ predictions.

#### Traveling waves approach

We derive an energy-velocity expansion of low-energy traveling wave solutions (1.5) with small velocities. To be more precise we consider states $( \psi , \varphi ) \in H^1( \mathbb {R}^3) \times L_{\sqrt{\varepsilon }}^2 ( \mathbb {R}^3)$

(i): with small energy, i.e. satisfying
  1.8 $$\begin{aligned} \mathcal {G}_\alpha ( \psi , \varphi ) \le e_\alpha + \kappa \; \text {for sufficiently small} \quad \kappa >0 \quad \text {(independent of }\; \alpha ) \end{aligned}$$
(ii)$$_\textrm{v}$$
and which are traveling wave solutions of velocity $\textrm{v}$, i.e. let $\textrm{v}< \textrm{v}_\textrm{crit}$ (uniformly in $\alpha$), then $( \psi _\textrm{v}, \varphi _\textrm{v})$ solves (1.5) with velocity $\textrm{v}$ and with phase $e_\textrm{v}\ge - e_\alpha + \textrm{v}^2/4$.

The definition of the effective mass through traveling waves is based on their energy-velocity expansion, i.e. for states of the set

1.9 $$\begin{aligned} \mathcal {I}_\textrm{v}:= \lbrace ( \psi , \varphi ) \in H^1( \mathbb {R}^3) \times L_{\sqrt{\varepsilon }}^2 ( \mathbb {R}^3) \vert \; \text {(i), (ii) }_\textrm{v}\text { are satisfied} \rbrace \; \end{aligned}$$

we study the energy expansion

1.10 $$\begin{aligned} E^\textrm{TW}_\textrm{v}:= \inf \lbrace \mathcal {G}_\alpha ( \psi , \varphi ) \vert \; ( \psi , \varphi ) \in \mathcal {I}_\textrm{v}\rbrace \; \end{aligned}$$

around the ground state energy $e_\alpha$.

##### Theorem 2

Let $\varepsilon , v$ satisfy Assumptions 1.1 and 1.2. Assume that for the pair of ground states $(\psi _\alpha , \varphi _\alpha )$ of $\mathcal {G}_\alpha$ given by (1.7) where $\varphi _\alpha = - \sqrt{\alpha } \sigma _{\psi _\alpha }$, the minimizer $\psi _\alpha$ is unique up to translations and changes of phase. There exists $\alpha _0 >0$ such that for all $\alpha \ge \alpha _0$ and $\alpha \textrm{v}\le 1$, we have

1.11 $$\begin{aligned} E_\textrm{v}^\textrm{TW} = e_\alpha + \left( m +\frac{ 2 (2\pi )^3 \alpha }{3} \Vert k v \varepsilon ^{-3/2} \; \widehat{\varrho }_{\psi _\alpha } \Vert _2^2 \right) \frac{\textrm{v}^2}{2} + O( \alpha \textrm{v}^3) \; . \end{aligned}$$

The energy expansion of Theorem 2 (i.e. (1.11)) is proven in Sect. 5.

The coefficients of the energy expansion are well defined as

1.12 $$\begin{aligned} \Vert k v \varepsilon ^{-3/2} \; \widehat{\varrho }_{\psi _\alpha } \Vert _2^2 = \Vert k v \varepsilon ^{-3/2} \; \widehat{\varrho }_{\widetilde{\psi }_\alpha } \Vert _2^2 \end{aligned}$$

for any

1.13 $$\begin{aligned} \psi _\alpha , \widetilde{\psi }_\alpha \in \Theta ( \psi _\alpha ) := \lbrace e^{i \omega } \psi _\alpha ^y =e^{i \omega } \psi _\alpha ( \cdot - y ) \vert \; y \in \mathbb {R}^3, \, \omega \in [0, 2 \pi ) \rbrace \; . \end{aligned}$$

We define the effective mass as the second order coefficient of the expansion of $E_\textrm{v}^\textrm{TW}$ around the ground state energy. It follows from the ground state’s approximation (see Proposition 2.2 below) that $\widehat{\varrho }_{\psi _\alpha } (k) \rightarrow 1$ point-wise in the limit $\alpha \rightarrow \infty$. Therefore in the strong coupling limit the leading order in $\alpha$ of the effective mass is given by

1.14 $$\begin{aligned} \lim _{\alpha \rightarrow \infty } \alpha ^{-1} m_\textrm{eff}^\textrm{TW} := \lim _{\alpha \rightarrow \infty } \alpha ^{-1} \left( \lim _{\textrm{v}\rightarrow 0} \frac{E_\textrm{v}^\textrm{TW} - e_\alpha }{\textrm{v}^2/2} \right) =\frac{ 2 (2\pi )^3 }{3} \Vert k v \varepsilon ^{-3/2} \; \Vert _2^2 \end{aligned}$$

and agrees with the findings of for the quantum Fröhlich model [18].

#### Approach through energy-momentum-expansion

For the second approach we are interested in the infimum of $\mathcal {G}_\alpha$ w.r.t. to the set of states $( \psi , \varphi ) \in H^1 ( \mathbb {R}^3) \times L^2_{\sqrt{\varepsilon }} ( \mathbb {R}^3 )$ with small energy (i.e. satisfy (i)) and

(ii)$$_\textrm{p}$$
with mean momentum $\textrm{p}\in \mathbb {R}^3$, i.e.
  1.15 $$\begin{aligned} \langle \widehat{\psi } \vert p \vert \widehat{\psi } \rangle + \langle \varphi |p|\varphi \rangle = \textrm{p}\; . \end{aligned}$$

Thus we consider states of the set

1.16 $$\begin{aligned} \mathcal {I}_{\textrm{p}}:= \lbrace (\psi , \varphi ) \in H^1( \mathbb {R}^3) \times L^2_{\sqrt{\varepsilon }} ( \mathbb {R}^3) \; \vert \; \text {such that (i),(ii)}_\textrm{p}\text { hold} \rbrace \; . \end{aligned}$$

The definition of the effective mass then relies on an expansion of

$$\begin{aligned} E_{\textrm{p}}:=\inf \lbrace \mathcal {G}_\alpha ( \psi , \varphi ) \vert \; \left( \psi , \varphi \right) \in \mathcal {I}_\textrm{p}\rbrace \end{aligned}$$

stated in the following theorem.

##### Theorem 3

Let $\varepsilon , v$ satisfy Assumptions 1.1 and 1.2. Assume that for the pair of ground states $(\psi _\alpha , \varphi _\alpha )$ of $\mathcal {G}_\alpha$ given by (1.7) where $\varphi _\alpha = - \sqrt{\alpha } \sigma _{\psi _\alpha }$, the minimizer $\psi _\alpha$ is unique up to translations and changes of phase.

- There exists $\alpha _0 >0$ such that for all $\alpha \ge \alpha _0$ and $\alpha ^{-1/4} \textrm{p}\le 1$
  1.17 $$\begin{aligned} E_\textrm{p}=&e_\alpha + \left( m +\frac{ 2 (2\pi )^3 \alpha }{3} \Vert k v \varepsilon ^{-3/2} \; \widehat{\varrho }_{\psi _\alpha } \Vert _2^2\right) ^{-1} \frac{\textrm{p}^2}{2} + O( \alpha ^{-5/4} \textrm{p}^3 ) \; . \end{aligned}$$
- There exists $\alpha _0 >0$ such that for all $\alpha \ge \alpha _0$ and $\alpha ^{-1/2}\textrm{p}\le 1$, a pair of minimizers $(\psi _\textrm{p},\varphi _\textrm{p})$ of $E_\textrm{p}$ is a traveling wave solution $( \psi _{\textrm{v}'}, \varphi _{\textrm{v}'} )$ to (4.1) with velocity $\textrm{v}' = m_\textrm{eff}^{-1} \textrm{p}+ O( \alpha ^{-3/2} \textrm{p}^2)$ and
  1.18 $$\begin{aligned} E_\textrm{p}= \mathcal {G}_\alpha ( \psi _{\textrm{v}'}, \varphi _{\textrm{v}'} ) + O ( \alpha ^{-2} \textrm{p}^3 ) \; . \end{aligned}$$

Theorem 3*(a), (b)* are proven in Sect. 5.

We define the effective mass as the coefficient of the second order contribution of the energy-momentum expansion and thus in leading order in $\alpha$ given in the strong coupling limit by

1.19 $$\begin{aligned} \lim _{\alpha \rightarrow \infty } \alpha ^{-1} m_\textrm{eff} :=&\lim _{\alpha \rightarrow \infty } \alpha ^{-1} \lim _{\textrm{p}\rightarrow 0 } \left( \frac{E_\textrm{p}- e_\alpha }{\textrm{p}^2/2} \right) ^{-1 } = \frac{ 2 (2\pi )^3 \alpha }{3} \Vert k v \varepsilon ^{-3/2} \Vert _2^2 \end{aligned}$$

which agrees with the effective mass $m_\textrm{eff}^\textrm{TW}$ defined in (1.14) and findings from the quantum Fröhlich model [18].

In particular Theorem 3*(b)* shows that any minimizer of $E_\textrm{p}$ is given by a traveling wave solution of velocity $\textrm{v}' = m_\textrm{eff}^{-1} \textrm{p}$, thus, by approximate elements of the set $\mathcal {I}_\textrm{v}$ considered in Theorem 2.

We remark that traveling wave solutions for non-linear Schrödinger equations with non-vanishing speed of sound were studied in various other settings (see for example [19] for the Gross-Pitaevksi and [20] for pseudo-relativistic Hartree equation). We note that [19] considers a variational approach to traveling waves in the spirit of Theorem 3*(b)*.

### Structure of the paper

In Sect. 2 we collect properties and approximations of the ground state, ground state energy (Sect. 2.1, Proposition 2.1 resp. Proposition 2.2) and traveling wave solutions (Sect. 2.2, Proposition 2.3) that will be important to prove our main theorems. In Sect. 3 we prove Propositions 2.1, 2.2 on the ground state’s properties. For this we first show the existence of ground states for all $\alpha >0$ in Sect. 3.1, then the approximation of the ground (state) by the harmonic oscillator in Sect. 3.2 and finally the positivity of the Hessian for large $\alpha > \alpha _0$ yielding coercivity estimates. We combine those results in Sect. 3.4 to finally prove Propositions 2.1, 2.2. In Sect. 4 we prove afterwards Proposition 2.3 (yielding in Theorem 1) on the properties of traveling waves. In Sect. 5 we finally prove Theorems 2 and 3 on the two definitions of the effective mass bases on the results before.

## Properties of the ground state and traveling waves

### Properties of the ground state

For the regularized polaron’s ground state

2.1 $$\begin{aligned} e_\alpha := \inf _{\psi ,\varphi } \mathcal {G}_\alpha ( \psi , \varphi ) \; \end{aligned}$$

the infimum can be taken first w.r.t. to the phonon field yielding by a completion of the square to the choice

2.2 $$\begin{aligned} \varphi _\alpha := - \sqrt{\alpha } \sigma _{\psi }, \quad \text {where} \quad \sigma _\psi := (2\pi )^{3/2} \frac{v}{ \varepsilon } \widehat{\varrho }_\psi \end{aligned}$$

with $\varrho _\psi = \vert \psi \vert ^2$. The resulting energy functional for $\psi \in H^1 ( \mathbb {R}^3)$ is

2.3 $$\begin{aligned} \mathcal {E}_\alpha ( \psi ) := \inf _{\varphi } \mathcal {G}_\alpha ( \psi , \varphi ) = \langle \psi |- \tfrac{\Delta }{2m} - \alpha \left( h* \vert \psi \vert ^2\right) |\psi \rangle \quad \textrm{with} \quad h= ( 2 \pi )^{3/2} \widehat{\left( v^2 / \varepsilon ^{-1} \right) } \; . \end{aligned}$$

Thus if $\psi _\alpha$ is an element of the manifold of minimizers $\mathcal {M}_{\mathcal {E}_\alpha }$ of the energy functional $\mathcal {E}_\alpha$ defined by

2.4 $$\begin{aligned} \mathcal {M}_{\mathcal {E}_\alpha } : = \lbrace \psi \in H^1 ( \mathbb {R}^3) \; \vert \Vert \psi \Vert _2=1, \; \mathcal {E}_\alpha ( \psi _\alpha ) = e_\alpha \rbrace \; , \end{aligned}$$

then the pair $( \psi _\alpha , \varphi _\alpha )$ with $\varphi _\alpha$ given by (2.2) is an element of the manifold of minimizers $\mathcal {M}_{\mathcal {G}_\alpha }$ of the energy functional $\mathcal {G}_\alpha$

2.5 $$\begin{aligned} \mathcal {M}_{\mathcal {G}_\alpha } := \lbrace ( \psi , \varphi ) \in H^1 ( \mathbb {R}^3) \times L^2_{\sqrt{\varepsilon }} ( \mathbb {R}^3) \; \vert \; \mathcal {G}_\alpha ( \psi , \varphi ) = e_\alpha \rbrace . \end{aligned}$$

The energy functional $\mathcal {E}_\alpha$ is symmetric with respect to translations and changes of the phase of the wave function. Thus for any minimizer $\psi _\alpha$ of $\mathcal {E}_\alpha$ (i.e. $\psi _\alpha \in \mathcal {M}_{\mathcal {E}_\alpha }$) it follows $\Theta ( \psi _\alpha ) \subseteq \mathcal {M}_{\mathcal {E}_\alpha }$ where

2.6 $$\begin{aligned} \Theta ( \psi _\alpha ) := \lbrace e^{i \omega } \psi _\alpha ^y =e^{i \omega } \psi _\alpha ( \cdot - y ) \vert \; y \in \mathbb {R}^3, \, \omega \in [0, 2 \pi ) \rbrace \; . \end{aligned}$$

For the (non-regularized) Pekar functional corresponding to (1.4), the existence of a unique pair of ground states $( \psi _\textrm{Pekar}, \varphi _\textrm{Pekar})$ up to phases and translations was proven [21] for all $\alpha >0$. For the regularized model we prove the existence of a ground state for all $\alpha >0$.

#### Proposition 2.1

(Existence) Let $\varepsilon , v$ satisfy Assumption 1.1. For all $\alpha >0$ there exists a pair of minimizers $( \psi _\alpha , \varphi _\alpha ) \in \mathcal {M}_{\mathcal {G}_\alpha }$ with $\varphi _\alpha = -\sqrt{\alpha } \sigma _{\psi _\alpha }$ and $0< \psi _\alpha \in C^\infty ( \mathbb {R}^3)$ satisfying the Euler-Lagrange equation

2.7 $$\begin{aligned} \left( \textrm{h}_{ \sqrt{\alpha }\varphi _\alpha } - \mu _{\psi _\alpha } \right) \psi _\alpha = 0, \quad \text {with} \quad \mu _{\psi _\alpha } := \langle \psi _\alpha |\textrm{h}_{\sqrt{\alpha }\varphi _\alpha }|\psi _\alpha \rangle \; . \end{aligned}$$

We remark that the ground state’s uniqueness for the regularized model is in general not known. For technical reasons, we can not prove uniqueness up to translations and phases for large $\alpha > \alpha _0$. For that, a refined approximation of the ground state than the one in Proposition 2.2 is needed to conclude ground state’s uniqueness up to translations and phase by the local coercivity estimates in Corollary 3.1 for large $\alpha > \alpha _0$.

Note that the first part of Theorems 2 and 3 immediately follow from Proposition 2.1 that is proven in Sect. 3.4.

The ground state $\psi _\alpha$’s properties for large coupling constants $\alpha >\alpha _0$ results from the asymptotic behavior of the energy functional $\mathcal {G}_\alpha$. In fact in the strong coupling limit $\alpha \rightarrow \infty$ the ground state energy $e_\alpha = \mathcal {G}_\alpha (\psi _\alpha )$ is well described through the harmonic oscillator

2.8 $$\begin{aligned} \mathrm{h_{osc}}:= - \frac{\Delta }{2m} + \frac{m \omega ^2x^2}{2} , \quad \text {with frequency} \quad \omega ^2 = \frac{ \alpha \Vert \nabla g\Vert _2^2}{3 m } \; . \end{aligned}$$

Furthermore its well known ground state

2.9 $$\begin{aligned} \psi _\textrm{osc}(x) := \left( \frac{m \omega }{ \pi }\right) ^{3/4} e^{-m\omega x^2/2} \end{aligned}$$

approximates the true ground state of $\mathcal {G}_\alpha$ as the following Lemma shows.

#### Proposition 2.2

(Approximation of the ground state) Let $\varepsilon ,v$ satisfy Assumption 1.1. There exists $\alpha _0 >0$ and constants $C_1,C_2 >0$ (independent of $\alpha$) such that

2.10 $$\begin{aligned} C_1 \alpha ^{1/3} \le e_\alpha + \alpha \Vert g\Vert _2^2 - \frac{\sqrt{3 \alpha } \Vert \nabla g\Vert _2^2}{2 \sqrt{m}} \le C_2 \; \quad \text {for all} \quad \alpha \ge \alpha _0 \; . \end{aligned}$$

Furthermore let $\psi _\alpha \in \mathcal {M}_{\mathcal {E}_\alpha }$. There exists $C_3>0$ (independent of $\alpha$) such that for all $\alpha \ge \alpha _0$

2.11 $$\begin{aligned} {{\,\textrm{dist}\,}}_{L^2} \left( \Theta ( \psi _\alpha ), \psi _\textrm{osc} \right) \le C_3 \alpha ^{-1/20}, \; \quad {{\,\textrm{dist}\,}}_{H^1} \left( \Theta ( \psi _\alpha ), \psi _\textrm{osc} \right) \le C_3 \alpha ^{9/40} \; . \end{aligned}$$

Proposition 2.2 is proven in Sect. 3.4.

Here we introduced the norm

2.12 $$\begin{aligned} {{\,\textrm{dist}\,}}_{L^2} \left( \Theta ( \psi _\alpha ) , \; \psi _\textrm{osc} \right) := \inf _{y', \theta '} \Vert e^{i \theta '}\psi _\alpha ^{y'} - \psi _\textrm{osc} \Vert _2 \end{aligned}$$

(and similarly for the $H^1$-norm) quantifying the distance of an element $\psi _\alpha$ of the manifold of minimizers to the harmonic oscillator’s ground state.

We remark that the rate of convergence of (2.11) depends for technical reasons on Assumption 1.1 namely the regularity of the function *g*.

### Traveling waves

The dynamical equations corresponding to the energy functional $\mathcal {G}_\alpha$ in (1.7) are given for $(\psi _t, \varphi _t ) \in H^1 (\mathbb {R}^3) \times L^2_{\sqrt{\varepsilon }} ( \mathbb {R}^3)$ by the system of coupled partial differential equations (1.2). The dynamical equations are well-posed as the following Lemma shows.

#### Lemma 2.1

Let $\varepsilon , v$ satisfy Assumption 1.1. For any $\left( \psi _0, \varphi _0 \right) \in H^1 ( \mathbb {R}^3) \times L_{\sqrt{\varepsilon }}^2 ( \mathbb {R}^3)$ there exists a unique global solution of (1.2). Furthermore,

2.13 $$\begin{aligned} \mathcal {G}_\alpha ( \psi _0,\varphi _0 ) = \mathcal {G}_\alpha ( \psi _t, \varphi _t ), \quad \text {and} \quad \Vert \psi _t \Vert _2 = \Vert \psi _0 \Vert _2 \end{aligned}$$

and there exists $C>0$ such that for $(\psi _0, \varphi _0)$ with $\mathcal {G}( \psi _0, \varphi _0) \le C \alpha$ we have for all $t \in \mathbb {R}$

2.14 $$\begin{aligned} \Vert \nabla \psi _t \Vert _2 \le C \sqrt{\alpha } \quad \text {and} \quad \Vert \varphi _t \Vert _{L^2_{\sqrt{\varepsilon }}} \le C \sqrt{\alpha } \; . \end{aligned}$$

The proof of the Lemma follows similarly to [5, Lemma 2.1] considering the non-regularized Landau-Pekar equations. The arguments presented in [5] apply for the regularized case, too, so that we refer for the proof of Lemma 2.1 to [5, Lemma 2.1].

A traveling wave of velocity $\textrm{v}\in \mathbb {R}^3$ is a solution of (1.2) with initial data $(\psi _\textrm{v}, \varphi _\textrm{v}) \in H^1( \mathbb {R}^3) \times L^2_{\sqrt{\varepsilon }} ( \mathbb {R}^3)$, $\Vert \psi _\textrm{v}\Vert _2 =1$ satisfying (1.5). The existence of subsonic traveling waves is given by the following Proposition.

#### Proposition 2.3

Let $\varepsilon , v$ satisfy Assumptions 1.1 and 1.2. Furthermore assume that $\vert \textrm{v}\vert < \textrm{v}_\textrm{crit}$.

- There exists a traveling wave solution of the form (1.5).
- Furthermore assume that $\mathcal {G}( \psi _\textrm{v}, \varphi _\textrm{v}) - e_\alpha \le \kappa$ for sufficiently small $\kappa >0$ (independent of $\alpha$) and $e_\textrm{v}\ge - e_\alpha + \textrm{v}^2/4$. Assume that the ground state $\psi _\alpha$ of $\mathcal {E}_\alpha$ is unique up to translations and rotations. Then there exists $\alpha _0 >0$ and a constant $C>0$ (independent of $\alpha , \textrm{v}$) such that
  2.15 $$\begin{aligned} {{\,\textrm{dist}\,}}_{L^2} \left( \Theta ( \psi _\alpha ) , \; \psi _\textrm{v}\right) \le C \vert \textrm{v}\vert \; \end{aligned}$$
  for all $\alpha \ge \alpha _0$ and $\vert \textrm{v}\vert \le 1$.

Note that Theorem 1 follows immediately from Proposition 2.3*(a)*. The proof of Proposition 2.3 is given in Sect. 2.2.

Furthermore note that a similar approximation as in (2.15) holds for the field $\varphi _\textrm{v}$, too. For this we remark that instead of minimizing w.r.t. to the field $\varphi$ in (2.1) first (as explained in Sect. 2.1) we can take the infimum w.r.t. to the wave function $\psi$ first, too yielding the functional

2.16 $$\begin{aligned} \mathcal {F}_\alpha ( \varphi ) = \inf _{\psi } \mathcal {G}_\alpha ( \psi , \varphi ), \quad \mathcal {M}_{\mathcal {F}_\alpha } = \lbrace \varphi \in L^2_{\sqrt{\varepsilon }} ( \mathbb {R}^3) \; \vert \mathcal {F}_\alpha ( \varphi _\alpha ) = e_\alpha \rbrace \; . \end{aligned}$$

We remark that by the energy functional’s symmetries for any $\varphi _\alpha \in \mathcal {M}_{\mathcal {F}_\alpha }$ and

2.17 $$\begin{aligned} \Omega (\varphi _\alpha ) := \lbrace e^{i z\cdot } \varphi _\alpha \vert \; z \in \mathbb {R}^3 \rbrace \end{aligned}$$

it follows that $\Omega ( \varphi _\alpha ) \subseteq \mathcal {M}_{\mathcal {F}_\alpha }$. Then under the same assumption as in Proposition 2.3 there exists $C>0$ (independent of $\alpha$) such that

2.18 $$\begin{aligned} {{\,\textrm{dist}\,}}_{L^2_{\sqrt{\varepsilon }}} \left( \Omega ( \varphi _\alpha ), \; \varphi _\textrm{v}\right) \le C \sqrt{\alpha } \vert \textrm{v}\vert \; . \end{aligned}$$

We remark that Proposition 2.3*(a)* shows that subsonic traveling waves exist for all $\alpha >0$. However the approximations (2.15), (2.18) of the second part of the Theorem holds for sufficiently large $\alpha \ge \alpha _0$ only. The restriction to sufficiently large $\alpha >0$ in part *(b)* ensures the validity of the global coercivity estimates (see Corollary 3.2) that are proven for sufficiently large $\alpha > \alpha _0$ only. Furthermore we notice that for $\textrm{v}=0$ the pair of ground states $( \psi _\alpha , \varphi _\alpha )$ provide a traveling wave solution with $e_\textrm{v}= e_\alpha$. In particular the assumption on the phase from part *(b)*, made for technical reasons only, is satisfied for $\textrm{v}=0$.

## Properties of the energy functional $\mathcal {E}_\alpha$

In this section, we prove Propositions 2.1 and 2.2 on the properties of the energy functional $\mathcal {G}_\alpha$.

The proof of Proposition 2.1 relies on a comparison of properties of $\mathcal {E}_\alpha$ with the properties of $\mathrm{h_{osc}}$ in the limit $\alpha \rightarrow \infty$. Then existence and uniqueness (up to translations and changes of the phase) for pairs of minimizers $( \psi _\alpha , \varphi _\alpha )$ of $\mathcal {G}_\alpha$ follow with the choice $\varphi _\alpha = - \sqrt{\alpha } \sigma _{\psi _\alpha }$.

First, in Lemma 3.1, we prove the existence of a minimizer $\psi _\alpha$ for all $\alpha$. Next we show the ground state (energy) is well approximated through the harmonic oscillator (Lemma 3.2). This approximations allows to show that the Hessian modulo its zero modes of $\mathcal {E}_\alpha$ is asymptotically for $\alpha \rightarrow \infty$ characterized by the harmonic oscillator, and thus positive (Lemma 3.3). This fact has several consequences: We infer first local (Corollary 3.1) and later global coercivity estimates (Corollary 3.2) for sufficiently large $\alpha \ge \alpha _0$. For the latter we assume that the ground state $\psi _\alpha$ of $\mathcal {M}_{\mathcal {E}_\alpha }$ is unique up to translations and phase. Furthermore we obtain that the ground state energy $e_\alpha$ is separated from the first excited eigenvalue by a gap of order $\sqrt{\alpha }$ (Corollary 3.3).

We remark that the strategy for the proofs in this section follow [6, Section 3] considering the non-regularized Pekar functional on a Torus of length *L*. For sufficiently large *L* the uniqueness of the ground state and coercivity estimates are proven based on a comparison with the non-regular Pekar functional defined on the full space for which these properties are well known.

### Existence

First we show the existence of minimizers of $\mathcal {E}_\alpha$ for all $\alpha >0$ in the subsequent Lemma.

#### Lemma 3.1

Let $\varepsilon , v$ satisfy Assumption 1.1. For all $\alpha >0$, there exists a minimizer $0< \psi _\alpha \in C^\infty ( \mathbb {R}^3)$ of the functional $\mathcal {E}_\alpha$ satisfying the Euler-Lagrange equation

3.1 $$\begin{aligned} \left( \textrm{h}_{ \sqrt{\alpha }\varphi _\alpha } - \mu _{\psi _\alpha } \right) \psi _\alpha = 0, \quad \text {with} \quad \mu _{\psi _\alpha } := \langle \psi _\alpha |h_{\sqrt{\alpha }\varphi _\alpha }|\psi _\alpha \rangle \; . \end{aligned}$$

#### Proof

Since $h = g* g$, we have $\Vert h \Vert _{\infty } \le C \Vert g \Vert _2^2$ and

3.2 $$\begin{aligned} \langle \psi |\left( h* \vert \psi \vert ^2\right) |\psi \rangle \le C \Vert g\Vert _2^2 \Vert \psi \Vert _2^4 \end{aligned}$$

so that by Assumption 1.1 there exists $C>0$ such that

3.3 $$\begin{aligned} \mathcal {E}_\alpha ( \psi ) \ge \tfrac{1}{2m} \Vert \nabla \psi \Vert _2^2 - C \alpha \; . \end{aligned}$$

From (3.3) we infer on one hand that $e_\alpha \ge -C \alpha$ for all $\alpha$. On the other hand, in order to prove the existence of a minimizer, we remark that (3.3) shows that any minimizing sequence $\left( \psi _n \right) _{n \in \mathbb {N}}$ is bounded in $H^1$, uniformly in $n \in \mathbb {N}$. For this reason the sequence by [22, Lemma 6] resp. [23, Theorem 8.10], there exists a sequence $\left( y_n \right) _{n \in \mathbb {N}} \in \mathbb {R}^3$ such that the translated sequence $\left( \psi _{n}^{y_n} \right) _{n \in \mathbb {N}}$ has a sub-sequence $\left( \psi _{n_j} \right) _{n_j \in \mathbb {N}}:= \left( \psi _{n_j}^{y_{n_j}} \right) _{n_j \in \mathbb {N}}$ that converges weakly in $H^1( \mathbb {R}^3)$ to a non-zero function. It follows from the Sobolev inequality that this sub-sequence converges strongly in $L^p$ for $2 \le p \le 6$ to a non-zero limit. The limiting function $\psi _\alpha \in H^1$ is again $L^2$-normalized and, moreover, satisfies

3.4 $$\begin{aligned} \langle \psi _\alpha |- \tfrac{\Delta }{2m}|\psi _\alpha \rangle \le \lim _{{n_j} \rightarrow \infty } \langle \psi _{n_j}|- \tfrac{\Delta }{2m}|\psi _{n_j}\rangle \end{aligned}$$

by semi-lower continuity of the $H^1$-norm. Since

3.5 $$\begin{aligned}&\left| \langle \psi _{n_j}|\left( h* \vert \psi _{n_j} \vert ^2 \right) |\psi _{n_j}\rangle - \langle \psi _\alpha |\left( h* \vert \psi _\alpha \vert ^2 \right) |\psi _\alpha \rangle \right| \nonumber \\&\quad \le \left| \left\langle \psi _{n_j}\right| \left( h* \vert \psi _{n_j} \vert ^2 \right) \left| \psi _\alpha - \psi _{n_j}\right\rangle \right| + \left| \left\langle \psi _\alpha \right| \left( h* \vert \psi _n \vert ^2 \right) \left| \psi _\alpha - \psi _{n_j}\right\rangle \right| \nonumber \\&\quad \quad + \left| \left\langle \psi _{n_j}\right| \left( h* \vert \psi _\alpha \vert ^2 \right) \left| \psi _\alpha - \psi _{n_j}\right\rangle \right| + \left| \left\langle \psi _\alpha \right| \left( h* \vert \psi _\alpha \vert ^2 \right) \left| \psi _\alpha - \psi _{n_j}\right\rangle \right| \end{aligned}$$

and $\Vert \left( h* \vert \psi _1 \vert ^2 \right) \psi _2 \Vert _{2} \le C \Vert \psi _1 \Vert _{2}^2 \Vert \psi _2 \Vert _{2}$ for any $\psi _1,\psi _2 \in L^2$, we find

3.6 $$\begin{aligned}&\left| \langle \psi _{n_j}|\left( h* \vert \psi _{n_j} \vert ^2 \right) |\psi _{n_j}\rangle - \langle \psi _\alpha |\left( h* \vert \psi _\alpha \vert ^2 \right) |\psi _\alpha \rangle \right| \le C \Vert \psi _\alpha - \psi _{n_j} \Vert _2 \rightarrow 0 \end{aligned}$$

as $$n \rightarrow \infty $$. Therefore,

3.7 $$\begin{aligned} \mathcal {E}_\alpha ( \psi _\alpha ) \le \liminf _{{n_j} \rightarrow \infty } \mathcal {E}_{\alpha } (\psi _{n_j} ) = e_\alpha \end{aligned}$$

and with $\mathcal {E}_ \alpha ( \psi _\alpha ) \ge \liminf _{{n_j} \rightarrow \infty } \mathcal {E}_\alpha ( \psi _{n_j}) = e_\alpha$ (as $( \psi _{n_j} )_{{n_j} \in \mathbb {N}}$ is a minimizing sequence), we conclude that $\mathcal {E}_{\alpha } ( \psi _\alpha ) = e_\alpha$ and, thus, $\psi _\alpha$ is a minimizer. By invariance of $\mathcal {E}_\alpha$ w.r.t. to translations and phase, any element of $\Theta (\psi _\alpha )$ defined in (2.6) is a minimizer, too. The positivity and regularity properties of $\psi _\alpha$ follows by standard bootstrap arguments (see for example [6, Lemma 3.3]). $\square$

### Approximation

Next we prove that in the strong coupling limit the spectrum of $\mathcal {E}_\alpha$ is well approximated by the harmonic oscillator $\mathrm{h_{osc}}$’s spectrum. The idea is to use the Taylor expansion of the potential given by Assumption 1.1 through

3.8 $$\begin{aligned} h( x) = \int \frac{v^2(k)}{\varepsilon (k)} e^{i k \cdot x} dk = \int \frac{v^2(k)}{\varepsilon (k)} \cos ( k \cdot x) dk \; \end{aligned}$$

to show its asymptotic quadratic behavior. The ground state energy of $\mathrm{h_{osc}}$ (as defined in (2.8)) is well known

3.9 $$\begin{aligned} e_\textrm{osc} = \frac{\sqrt{3\alpha }\Vert \nabla g \Vert _2^2}{ 2\sqrt{m}} \end{aligned}$$

and separated from the rest of the spectrum by a gap of order $\sqrt{\alpha }$. For this we compare the Hessian of $\mathcal {E}_\alpha$ with

3.10 $$\begin{aligned} \mathcal {H}_\textrm{osc}:= \inf _{\begin{array}{c} f \in H^1 ( \mathbb {R}^3), \Vert f \Vert _2 =1 \\ f \in \textrm{span} \lbrace \psi _\textrm{osc}\rbrace ^\perp \end{array}} \langle f|\mathrm{H_{osc}}|f\rangle , \quad \text {with} \quad \mathrm{H_{osc}}= \mathrm{h_{osc}}- e_\textrm{osc} \end{aligned}$$

that is known to be positive, and thus, yielding coercivity estimates of the form

3.11 $$\begin{aligned} \langle f|\mathrm{h_{osc}}|f\rangle - e_\textrm{osc}&\ge C \alpha ^{1/2} \inf _{\theta \in (0, 2 \pi ]}\Vert e^{i \theta } \psi _\textrm{osc}- f \Vert _2^2 \nonumber \\ \langle f|\mathrm{h_{osc}}|f\rangle - e_\textrm{osc}&\ge C \alpha ^{1/4} \inf _{\theta \in (0, 2 \pi ]}\Vert e^{i \theta } \psi _\textrm{osc}- f \Vert _{H^1 ( \mathbb {R}^3)}^2 \; . \end{aligned}$$

Furthermore we compare the deviation of the ground state of $\psi _\alpha$ with the one of the harmonic oscillator that is known to be $\Vert x^2 \psi _\textrm{osc}\Vert _2 = C \alpha ^{-1/2}$ for some $C>0$.

#### Lemma 3.2

Let $\varepsilon , v$ satisfy Assumption 1.1.

- Then there exists $C_1,C_2,C_3>0$ (independent of $\alpha$) such that
  3.12 $$\begin{aligned} C_1 \alpha ^{1/3} \le e_\alpha + \alpha \Vert g\Vert _2^2 - \frac{\sqrt{3 \alpha } \Vert \nabla g\Vert _2^2}{2 \sqrt{m}} \le C_2 \; \end{aligned}$$
  and
  3.13 $$\begin{aligned} \vert \mu _{\psi _\alpha } - \mu _{\psi _\textrm{osc}} \vert \le C_3 \alpha ^{5/12} \; \end{aligned}$$
  where we introduced the notation $\mu _{\psi _\textrm{osc}} = \langle \psi _\textrm{osc}|- \Delta + 2 \alpha (h * \vert \psi _\textrm{osc}\vert ^2)|\psi _\textrm{osc}\rangle$. Let $\psi _\alpha \in \mathcal {M}_{\mathcal {E}_\alpha }$ such that
  3.14 $$\begin{aligned} {{\,\textrm{dist}\,}}_{L^2} \left( \Theta ( \psi _\alpha ), \psi _\textrm{osc} \right) = \Vert \psi _\textrm{osc}- \psi _\alpha \Vert _2 \; . \end{aligned}$$
  Then there exists $\alpha _0 >0$ and $C>0$ (independent of $\alpha$) such that for all $\alpha \ge \alpha _0$ we have
  3.15 $$\begin{aligned} \Vert x^2 \psi _\alpha \Vert _2 \le C \alpha ^{-1/2} \end{aligned}$$
- Let $\psi _\alpha \in \mathcal {M}_{\mathcal {E}_\alpha }$. Then there exists $\alpha _0 >0$ and $C>0$ (independent of $\alpha$) such that for all $\alpha \ge \alpha _0$ we have
  3.16 $$\begin{aligned} {{\,\textrm{dist}\,}}_{L^2} \left( \Theta ( \psi _\alpha ), \psi _\textrm{osc} \right) \le C \alpha ^{-1/20} \; . \end{aligned}$$
- Let $\psi _\alpha \in \mathcal {M}_{\mathcal {E}_\alpha }$ such that
  3.17 $$\begin{aligned} {{\,\textrm{dist}\,}}_{L^2} ( \Theta ( \psi _\alpha ), \psi _\textrm{osc}) = \Vert \psi _\alpha - \psi _\textrm{osc}\Vert _2 \; . \end{aligned}$$
  Then there exists $\alpha _0 >0$ and $C>0$ (independent of $\alpha$) such that for sufficiently large $\alpha \ge \alpha _0$ we have
  3.18 $$\begin{aligned} \Vert x^2 \left( \psi _\alpha - \psi _\textrm{osc}\right) \Vert _2 \le C \alpha ^{-11/20} \; . \end{aligned}$$
- Under the same assumptions as in part (c) there exists $\alpha _0 >0$ and $C_1,C_2,C_3 >0$ (independent of $\alpha$) such that for sufficiently large $\alpha \ge \alpha _0$ we have
  3.19 $$\begin{aligned} C_1 \alpha ^{1/4} \le \Vert \nabla \psi _\alpha \Vert _2 \le C_2 \alpha ^{1/4} \; \end{aligned}$$
  and furthermore
  3.20 $$\begin{aligned} \Vert \nabla \left( \psi _\alpha - \psi _\textrm{osc}\right) \Vert _2 \le C_3 \alpha ^{9/40} \; . \end{aligned}$$

#### Proof

First we remark that in the following proof we denote with $C>0$ a constant independent of $\alpha$.

**Proof of** *(a)***:** For the upper bound of the ground state $e_\alpha$ we pick the harmonic oscillator’s ground state $\psi _\textrm{osc}$ defined in (2.9) as trial state. Its energy serves as an upper bound for the ground state energy

3.21 $$\begin{aligned} e_\alpha \le \mathcal {E}_\alpha \left( \psi _\textrm{osc}\right) \; \end{aligned}$$

and can be explicitly computed with by the potential’s (3.8) Taylor expansion, Assumption 1.1 and $\cos ( x) \ge 1 - \frac{x^2}{2}$

3.22 $$\begin{aligned} e_\alpha \le - \alpha \Vert g \Vert _2^2 + e_\textrm{osc} + C \end{aligned}$$

for a constant $C >0$ (independent of $\alpha$).

For the lower bound we use the IMS localization technique to show that it suffices to consider the problem on a ball of radius *R*, where we can use the potential’s Taylor expansion. To this end, let $\psi _\alpha$ denote a minimizer realizing

3.23 $$\begin{aligned} e_\alpha = \inf _{\psi \in H^1( \mathbb {R}^3)} \mathcal {E}_\alpha ( \psi ) = \mathcal {E}_\alpha ( \psi _\alpha ) \; \end{aligned}$$

and $\chi \in C^\infty ( \mathbb {R}^3)$ a function with support on the ball $B_1$ with radius one such that $\Vert \chi \Vert =1$ and $\chi (0) =1$. We define the rescaled function $\chi ^R$ with $\Vert \chi ^R \Vert _2 =1$ supported on $B_R$ and denote with $\chi ^{R,z} = \chi ^R ( \cdot - z)$ its shift. The idea is to choose *R* dependent on $\alpha$. However for simplicity we neglect the dependence of *R* on $\alpha$ in the notation. We observe that the $L^2$-normalized function

3.24 $$\begin{aligned} \psi ^{R,z}_\alpha = \chi ^{R,z} \psi _\alpha / \Vert \chi ^{R,z} \psi _\alpha \Vert _2 \end{aligned}$$

satisfies

3.25 $$\begin{aligned} \vert \psi _\alpha (x) \vert ^2 = \int _{B_R} \vert \chi ^{R,z} \psi _\alpha \vert ^2 (x) dz = \int _{B_R} \vert \psi ^{R,z}_\alpha (x) \vert ^2 \Vert \chi ^{R,z} \psi _\alpha \Vert _2^2 dz \end{aligned}$$

and thus, by completing the square and standard techniques of IMS localization

3.26 $$\begin{aligned} \mathcal {E}_\alpha ( \psi _\alpha ) + \Vert \nabla \chi ^{R}\Vert _2^2 = \int _{B_R} \left( \mathcal {E}_\alpha ( \psi ^{R,z}_\alpha ) + \alpha \Vert v \varepsilon ^{-1/2} ( \widehat{\varrho }_{\psi _\alpha } - \widehat{\varrho }_{\psi ^{R,z}_\alpha } ) \Vert _2^2 \right) \Vert \chi ^{R,z} \psi _\alpha \Vert _2^2 dz \; . \end{aligned}$$

Since $\chi ^{R,z} \psi _\alpha \Vert _2^2 dz$ denotes a probability measure, there exists $z \in B_R$ such that

3.27 $$\begin{aligned} \mathcal {E}_\alpha ( \psi _\alpha ) + \Vert \nabla \chi ^{R}\Vert _2^2 \ge \mathcal {E}_\alpha ( \psi ^{R,z}_\alpha ) + \alpha \Vert v \varepsilon ^{-1/2} ( \widehat{\varrho }_{\psi _\alpha } - \widehat{\varrho }_{\psi ^{R,z}_\alpha } ) \Vert _2^2 \; . \end{aligned}$$

By scaling, we furthermore find $\Vert \nabla \chi ^{R} \Vert _2^2 \le C R^{-2}$ yielding

3.28 $$\begin{aligned} \mathcal {E}_\alpha ( \psi _\alpha ) \ge \mathcal {E}_\alpha ( \psi ^{R,z}_\alpha ) + \alpha \Vert v \varepsilon ^{-1/2} ( \widehat{\varrho }_{\psi _\alpha } - \widehat{\varrho }_{\psi ^{R,z}_\alpha } ) \Vert _2^2 - CR^{-2} \; . \end{aligned}$$

We use (3.28) to prove both, the energy’s lower bound and the approximation of the ground state. We start with the lower bound on the ground state energy first. For this, we observe that (3.28) implies

3.29 $$\begin{aligned} \mathcal {E}_\alpha ( \psi _\alpha ) \ge \mathcal {E}_\alpha ( \psi ^{R,z}_\alpha ) - CR^{-2} \; , \end{aligned}$$

i.e. it suffices to compute the energy $\mathcal {E}_\alpha$ for function $\psi ^{R,z}_\alpha$ supported on $B_R$, where we can use the Taylor expansion of $h$

3.30 $$\begin{aligned} \mathcal {E}_\alpha ( \psi _\alpha ^{R,z} )&= \langle \psi _\alpha ^{R,z}|- \frac{\Delta }{2m} + \alpha ( h* \vert \psi _{\alpha }^{R,z} \vert ^2 )| \psi _\alpha ^{R,z}\rangle \nonumber \\&\ge - \alpha \Vert g \Vert _2^2 + \langle \psi _\alpha ^{R,z}|- \frac{\Delta }{2m} + \alpha \Vert \nabla g \Vert _2^2 ( x^2 * \vert \psi _\alpha ^{R,z} \vert ^2 )| \psi _\alpha ^{R,z}\rangle - C \alpha R^4 \; . \end{aligned}$$

We observe that (3.27) is invariant w.r.t. translations and changes of phase of $\psi _\alpha$ and $\psi ^{R,z}$ and thus, we can furthermore restrict to $\psi _\alpha ^{R,z}$ such that

3.31 $$\begin{aligned} \langle \psi _\alpha ^{R,z}|x|\psi _\alpha ^{R,z}\rangle =0 \end{aligned}$$

for which we find

3.32 $$\begin{aligned} \mathcal {E}_\alpha ( \psi _\alpha ) \ge \langle \psi ^{R,z}_\alpha |\textrm{h}_\textrm{osc}| \psi ^{R,z}_\alpha \rangle - \alpha \Vert g \Vert _2^2- C \alpha R^4 - CR^{-2} \; . \end{aligned}$$

where $\mathrm{h_{osc}}$ denotes the harmonic oscillator $\textrm{h}_\textrm{osc} = - \frac{\Delta }{2\,m} + \frac{m\omega ^2}{2} x^2$. By definition, $\psi ^{R,z}_\alpha$ is $L^2$-normalized and thus a competitor for the ground state of $\mathrm{h_{osc}}$, i.e.

3.33 $$\begin{aligned} \mathcal {E}_\alpha ( \psi _\alpha )&\ge \inf _{\psi \in H^1}\langle \psi |\textrm{h}_\textrm{osc}| \psi \rangle - \alpha \Vert g \Vert _2^2 - C \alpha R^4 - CR^{-2} \nonumber \\&\ge e_\textrm{osc} - \alpha \Vert g \Vert _2^2 - C \alpha R^4 - CR^{-2} \; . \end{aligned}$$

Optimizing w.r.t. to the parameter *R* (yielding $R= \alpha ^{-1/6}$), we arrive at

3.34 $$\begin{aligned} e_\alpha \ge e_\textrm{osc} - \alpha \Vert g \Vert _2^2 - C \alpha ^{1/3} \; \end{aligned}$$

proving part *(a)*.

*Properties of* $\psi _\alpha , \psi _\alpha ^{R,z}$*:* As a preliminary step to prove the remaining parts of this Proposition we prove useful properties of the ground state $\psi _\alpha$ and $\psi _\alpha ^{R,z}$ (constructed in (3.24), satisfying (3.27) and by translational invariance of the problem (3.31)). We observe that $\cos ( x ) \le 1$ (and thus $h \le \Vert g \Vert _2^2$) implies

3.35 $$\begin{aligned} \langle \psi _\alpha ^{R,z}|- \tfrac{\Delta }{2m}| \psi _\alpha ^{R,z}\rangle = \mathcal {E}_\alpha (\psi _\alpha ^{R,z} ) + \alpha \langle \psi _\alpha ^{R,z}|(h* \vert \psi _{\alpha }^{R,z}\vert ^2)|\psi _\alpha ^{R,z}\rangle \le \mathcal {E}_\alpha (\psi _\alpha ^{R,z} ) + \alpha \Vert g \Vert _2^2 \; . \end{aligned}$$

With(3.28) and the ground state energy’s approximation (part *(a)*) we obtain

3.36 $$\begin{aligned} \Vert \psi _\alpha ^{R,z} \Vert _{H^1}^2 \le C \sqrt{\alpha } , \quad \text {and similarly} \quad \Vert \psi _\alpha \Vert _{H^1}^2 \le C \sqrt{\alpha } \; , \end{aligned}$$

and in the same way

3.37 $$\begin{aligned} \alpha \Vert v/\varepsilon ^{1/2} \widehat{\varrho }_{\psi _\alpha } \Vert _2^2, \; \alpha \Vert v/\varepsilon ^{1/2} \widehat{\varrho }_{\psi _\alpha ^{R,z}} \Vert _2^2 \le C \alpha \; . \end{aligned}$$

With the upper bound (3.34) we furthermore deduce from (3.35) resp. the Euler-Lagrange equation

3.38 $$\begin{aligned} \Vert \Delta \psi _\alpha \Vert _2^2 \le C \sqrt{\alpha } \end{aligned}$$

for all $\alpha \ge \alpha _0$. However for $\psi _{\alpha }^{R,z}$ we observe first that (3.28) resp. (3.32) together with the harmonic oscillator’s coercivity property (3.11) show for $R= \alpha ^{-1/6}$

3.39 $$\begin{aligned} \sqrt{\alpha } \inf _{\theta }\Vert \psi _\alpha ^{R,z} - e^{i \theta }\psi _\textrm{osc}\Vert _2^2&\le \langle \psi _\alpha ^{R,z} |\textrm{h}_\textrm{osc}| \psi _\alpha ^{R,z} \rangle - e_\textrm{osc} \nonumber \\&\le \mathcal {E}_\alpha ( \psi _\alpha ^{R,z} ) - e_\textrm{osc}+ \alpha \Vert g \Vert _2^2 + C \alpha ^{1/3} \; . \end{aligned}$$

With the upper bound on the energy (3.22) we find

3.40 $$\begin{aligned} \inf _{\theta }\Vert \psi ^{R,z}_\alpha - e^{i \theta } \psi _\textrm{osc}\Vert _2^2 \le C \alpha ^{-1/6}, \quad \inf _{\theta }\Vert \psi ^{R,z}_\alpha - e^{i \theta } \psi _\textrm{osc}\Vert _{H^1}^2 \le C \alpha ^{1/12} \; \, . \end{aligned}$$

In particular for sufficiently large $\alpha \ge \alpha _0$ we have

3.41 $$\begin{aligned} \Vert \nabla \psi _\alpha ^{R,z} \Vert _2 \le C \alpha ^{-1/4} \; . \end{aligned}$$

*Approximation of Lagrange multipliers:* We prove the approximation of the Lagrange multiplier $\mu _{\psi _\alpha }$ with $\mu _{\psi _\textrm{osc}}$ using the previous results. In particular by translational invariance of the problem we choose $\psi _\alpha$ such that $\psi _\alpha ^{R,z}$ (as constructed in (3.24)) satisfies (3.27) and (3.31)). We write

3.42 $$\begin{aligned} \mu _{\psi _\alpha } - \mu _{\psi _\textrm{osc}} = \mathcal {E}_\alpha ( \psi _\alpha ) - \mathcal {E}_\alpha ( \psi _\textrm{osc}) - \alpha \Vert v / \varepsilon ^{1/2} \varrho _{\psi _\alpha } \Vert _2^2 + \alpha \Vert v / \varepsilon ^{1/2} \varrho _{\psi _\textrm{osc}} \Vert _2^2 \end{aligned}$$

yielding with (3.27) and (3.37) to

3.43 $$\begin{aligned} \vert \mu _{\psi _\alpha } - \mu _{\psi _\textrm{osc}} \vert \le&\vert \mathcal {E}_\alpha ( \psi _\alpha ) - \mathcal {E}_\alpha ( \psi _\textrm{osc}) \vert \nonumber \\&+\alpha \left( \Vert v / \varepsilon ^{1/2} \varrho _{\psi _\textrm{osc}} \Vert _2 + \Vert v / \varepsilon ^{1/2} \varrho _{\psi _\alpha } \Vert _2 \right) \Vert v / \varepsilon ^{1/2} \left( \varrho _{\psi _\alpha } -\varrho _{\psi _\textrm{osc}} \right) \Vert _2 \nonumber \\ \le&C \alpha ^{1/3} + \sqrt{\alpha }\Vert v / \varepsilon ^{1/2} \left( \varrho _{\psi _\alpha } -\varrho _{\psi _\textrm{osc}} \right) \Vert _2 \; . \end{aligned}$$

Since

3.44 $$\begin{aligned} \Vert v / \varepsilon ^{1/2} ( \varrho _{\psi _\alpha } -\varrho _{\psi _\textrm{osc}} ) \Vert _2 \le \Vert v / \varepsilon ^{1/2} (\varrho _{\psi _\alpha ^{R,z}} -\varrho _{\psi _\textrm{osc}} ) \Vert _2 + \Vert v / \varepsilon ^{1/2} ( \varrho _{\psi _\alpha ^{R,z}} -\varrho _{\psi _\textrm{osc}} ) \Vert _2 \end{aligned}$$

we find with (3.27), $\Vert v / \varepsilon ^{1/2} ( \varrho _{\psi _\alpha ^{R,z}} -\varrho _{\psi _\textrm{osc}}) \Vert _2 \le C \Vert \psi _\textrm{osc}- \psi _\alpha ^{R,z}\Vert _2$ and (3.40)

3.45 $$\begin{aligned} \Vert v / \varepsilon ^{1/2} \left( \varrho _{\psi _\alpha } -\varrho _{\psi _\textrm{osc}} \right) \Vert _2 \le C \alpha ^{-1/3} + C \alpha ^{-1/12} \; . \end{aligned}$$

Thus we obtain from (3.43) for sufficiently large $\alpha >\alpha _0$

3.46 $$\begin{aligned} \vert \mu _{\psi _\alpha } - \mu _{\psi _\textrm{osc}} \vert \le&C \alpha ^{5/12} \; . \end{aligned}$$

*Scaling of the ground state:* To show the ground state’s scaling (3.15) for $\psi _\alpha$ satisfying (3.14) we observe that by the Euler-Lagrange equation we have

3.47 $$\begin{aligned} \left( - \Delta - \mu _{\psi _\alpha } \right) \psi _\alpha = 2 \alpha \left( h * \vert \psi _\alpha \vert ^2 \right) \psi _\alpha \; . \end{aligned}$$

The idea is now to use the properties of the resolvent $\left( - \Delta - \mu _{\psi _\alpha } \right) ^{-1}$ (that is well defined sind from (3.46) we have $\mu _{\psi _\alpha } \ge - C \alpha$ for sufficiently large $\alpha \ge \alpha _0$) to prove the desired bound. The resolvent’s Green’s function is given in terms of the inverse Fourier transform $\mathcal {F}^{-1}$ of

3.48 $$\begin{aligned} G_{\mu _{\psi _\alpha }} (z) = \mathcal {F}^{-1} \left[ \big ( p^2 /(2m) - \mu _{\psi _\alpha } \big )^{-1} \right] (z) \end{aligned}$$

and can by functional calculus explicitly computed. In fact we have

3.49 $$\begin{aligned} \mathcal {F}\left[ \left( - \tfrac{1}{2m}\Delta - \mu _{\psi _\alpha } \right) ^{-1} \right] (p) = \int _0^\infty e^{- t (\tfrac{1 }{2m} p^2 - \mu _{\psi _\alpha } ) } dt \end{aligned}$$

which leads with (3.48) to

3.50 $$\begin{aligned} G_{ \mu _{\psi _\alpha }} (z)&= \frac{1}{\left( 2 \pi \right) ^{3/2}} \int _{\mathbb {R}^3} \int _0^\infty e^{i z \cdot p} e^{t \mu _{\psi _\alpha }} e^{-\tfrac{t}{2m} p^2} dt dp \end{aligned}$$

and we arrive with Fubini’s theorem at

3.51 $$\begin{aligned} G_{\mu _{\psi _\alpha }} (z) = (2m)^{3/2} \int _0^\infty e^{t \mu _{\psi _\alpha } } \frac{e^{-\frac{mz^2}{2t}}}{ t^{3/2} } dt = (2m)^{3/2} \frac{\sqrt{\pi }}{\vert z \vert } e^{-\sqrt{-4m \mu _{\psi _\alpha }} \vert z \vert } \end{aligned}$$

for $\vert z \vert \not =0$. We plug this identity into (3.47) and find using assumption (3.14), and the notation $F: = 2 \alpha \left( h * \vert \psi _\alpha \vert ^2 \right) \psi _\alpha$ (i.e. *F* denotes the right hand side of (3.47))

3.52 $$\begin{aligned} \psi _\alpha (x) = \int G_{\mu _{\psi _\alpha }} (x-y) \; F(y) dy \; . \end{aligned}$$

Thus the weighted $L^2$-norm, we aim to find an upper bound for, becomes

3.53 $$\begin{aligned} \Vert x^{3} \psi _\alpha \Vert _2^2 =&\int x^{6} G_{\mu _{\psi _\alpha }} (x-y)G_{\mu _{\psi _\alpha }} (x-z) \; F(z) F(y) \; dxdydz \end{aligned}$$

that we can estimate by Cauchy Schwarz with

3.54 $$\begin{aligned} \Vert x^{3}\psi _\alpha \Vert _2^2 \le&\int x^{6} \; \vert G_{\mu _{\psi _\alpha }} (x-y) \vert ^2 \vert F(y) \vert ^2 dxdy \nonumber \\ =&(2m)^3 \pi \int \frac{ x^{6}}{ \vert x -y \vert ^2 } e^{-2 \sqrt{-4m \mu _{\psi _\alpha } } \vert x-y \vert } \vert F(y) \vert ^2 dydx \; . \end{aligned}$$

We split the integral into several regions to find the desired bound: First we consider the case $\vert x \vert > 2 \vert y \vert$ for which we have $\vert x - y\vert \ge \vert x \vert - \vert y \vert \ge \frac{\vert x \vert }{2}$. By substitution we can therefore estimate the integral in this region by

3.55 $$\begin{aligned}&\int _{\vert x \vert > 2 \vert y \vert } \frac{ x^{6}}{\vert x- y\vert ^2} \; e^{-2 \sqrt{-4m \mu _{\psi _\alpha } } \vert x-y \vert } \vert F(y) \vert ^2 dydx \nonumber \\&\quad \le \int \; x^{4} e^{-2 \sqrt{-4m \mu _{\psi _\alpha } } \vert x \vert } \vert F(y) \vert ^2 dydx \le \frac{C }{ \alpha ^{5/2}} \Vert F \Vert _2^2 \end{aligned}$$

where we used $- \mu _{\psi _\alpha } \ge C \alpha$ for sufficiently large $\alpha \ge \alpha _0$. Now let $\vert x \vert <2 \vert y \vert$ and $\vert x-y\vert > \frac{\vert y \vert }{2}$, then we have $\frac{\vert x \vert }{\vert x-y\vert } \le 4$ and it follows

3.56 $$\begin{aligned}&\int _{\begin{array}{c} \vert x \vert < 2 \vert y \vert \\ \vert x-y\vert \ge \vert y \vert /2 \end{array}} \frac{ x^{6} }{\vert x- y\vert ^2}\;e^{-2 \sqrt{-4m \mu _{\psi _\alpha } } \vert x-y \vert } \vert F(y) \vert ^2 dydx \nonumber \\&\quad \le 4 \int x^{4} e^{- \sqrt{-4m \mu _{\psi _\alpha } } \vert y \vert } \vert F(y) \vert ^2 dydx \le \frac{C }{ \alpha ^{5/2}}\Vert F \Vert _2^2 \; . \end{aligned}$$

with similar arguments as before. Finally for $\vert x \vert < 2 \vert y \vert$ and $\vert x-y\vert \le \frac{\vert y \vert }{2}$ it follows $\vert x \vert \ge \vert y \vert - \vert x - y \vert \ge \vert y \vert /2$. Hence

3.57 $$\begin{aligned}&\int _{\begin{array}{c} \vert x \vert< 2 \vert y \vert \\ \vert x-y\vert< \vert y \vert /2 \end{array}} \frac{ x^{6}}{\vert x- y\vert ^2} \; e^{-2 \sqrt{-4m \mu _{\psi _\alpha } } \vert x-y \vert } \vert F(y) \vert ^2 dydx \nonumber \\&\quad \le 2 \int _{ \vert y \vert /2< \vert x \vert < 2 \vert y \vert } \frac{ y^{6}}{\vert x-y\vert ^2} e^{- \sqrt{-4m \mu _{\psi _\alpha } } \vert x- y \vert } \vert F(y) \vert ^2 dydx \nonumber \\&\quad \le \frac{C}{( -\mu _{\psi _\alpha } )^{1/2}} \int y^{6} e^{- C\sqrt{-4m \mu _{\psi _\alpha } } \vert y \vert } \vert F(y) \vert ^2 dy \le \frac{C }{ \alpha ^{5/2}} \Vert F\Vert _2^2 \end{aligned}$$

where we used that $\vert y \vert ^{6} \; e^{- C \sqrt{-4\,m \mu _{\psi _\alpha }} \vert y\vert } \le \frac{\widetilde{C}}{ \alpha ^{3}}$. Summarizing the estimates we conclude by

3.58 $$\begin{aligned} \Vert x^3 \psi _\alpha \Vert _2 \le C \alpha ^{-5/4 }\Vert F \Vert _2 \; \end{aligned}$$

where *F* denotes the r.h.s. of (3.47). Since $\Vert h \Vert _\infty \le C$,$\psi _\alpha$ is a $L^2$-normalized function we find that

3.59 $$\begin{aligned} \Vert x^{3} \psi _\alpha \Vert _2 \le C \alpha ^{-3/4} \; . \end{aligned}$$

for sufficiently large $\alpha \ge \alpha _0$. In particular for $n=3$ we find $\Vert x^3 \psi _\alpha \Vert _2 \le C \alpha ^{-3/4}$ and thus, in particular,

3.60 $$\begin{aligned} \Vert x^2 \psi _\alpha \Vert _2^2 \le C \alpha ^{-1/2}. \end{aligned}$$

**Proof of** *(b)***:** In order to prove the ground state $$\psi _\alpha $$’s approximation we observe that by the previous discussion it is enough to consider the problem on the ball $$B_{R_1} (0) $$ with $$R_1 = \alpha ^{-1/5}$$. In fact (3.15) shows

3.61 $$\begin{aligned} \inf _{\theta } \Vert \psi _\textrm{osc}- e^{i \theta } \psi _\alpha \Vert _2 \le \Vert \psi _\textrm{osc}- e^{i \theta } \psi _\alpha \Vert _{ L^2( B_{R_1} (0)) } + C \alpha ^{-1/20} \; . \end{aligned}$$

We consider $\psi _\alpha$ and $\psi _\alpha ^{R,z}$ (constructed in (3.24)), satisfying (3.27) and by translational invariance of the problem (3.31)). With these notations we in particular have from (3.40)

3.62 $$\begin{aligned} \inf _{\theta } \Vert \psi _\textrm{osc}- e^{i \theta } \psi _\alpha \Vert _{ L^2( B_{R_1} (0)) }&\le \Vert \psi _\alpha - \psi _\alpha ^{R,z} \Vert _{ L^2( B_{R_1} (0)) } + \inf _{\theta } \Vert \psi _\textrm{osc}- e^{i \theta } \psi _\alpha ^{R,z} \Vert _{ L^2( B_{R_1} (0)) } \nonumber \\&\le \Vert \psi _\alpha - \psi _\alpha ^{R,z} \Vert _{ L^2( B_{R_1} (0)) } + C \alpha ^{-1/12} \end{aligned}$$

and thus it remains to show $\psi _\alpha ^{R,z}$ is close to $\psi _\alpha$. To this end we control the $L^2$-norm of the difference $\varrho _{\psi _\alpha } - \varrho _{\psi ^{R,z}_\alpha }$ first and deduce as a second step from this estimates for $L^2$-norm of $\psi _\alpha ^{R,z} -\psi _\alpha$. For this we write the $L^2$-norm of $\varrho _{\psi _\alpha } - \varrho _{\psi ^{R,z}_\alpha }$ in momentum space. We shall show that for low momenta, we control the norm with Assumption 1.1 and (3.27) while for high momenta the $L^2$-norm is small by regularity properties of $\varrho _{\psi _\alpha },\varrho _{\psi ^{R,z}_\alpha }$. For the latter one we observe that from (3.38) we have

3.63 $$\begin{aligned} \Vert k^2 \widehat{\varrho }_{\psi _\alpha } \Vert _\infty \le C \Vert \Delta \psi _\alpha \Vert _2 \Vert \psi _\alpha \Vert _2 + C \Vert \nabla \psi _\alpha \Vert _2^2 \le C \alpha ^{1/2} \; \end{aligned}$$

and thus there exists $C>0$ such that

3.64 $$\begin{aligned} \vert \widehat{\varrho }_{\psi _\alpha } (k) \vert \le \frac{C \alpha ^{1/2}}{\vert k \vert ^2} \, , \end{aligned}$$

To obtain a similar bound for $\widehat{\varrho }_{\psi _\alpha ^{R,z}}$ we first have to derive a bound for the $H^2$-norm of $\psi _\alpha ^{R,z}$. For this we remark that by definition (3.24) we have

3.65 $$\begin{aligned} \Vert \chi ^{R,z} \psi _\alpha \Vert _2 \Delta \psi _\alpha ^{R,z} = \chi ^{R,z} ( \Delta \psi _\alpha ) + 2 ( \nabla \chi ^{R,z}) ( \nabla \psi _\alpha ) + ( \Delta \chi ^{R,z} ) \psi _\alpha \; . \end{aligned}$$

With the Taylor expansion of $\chi ^{R,z}$ around *z* we find that $\Vert \chi ^{R,z} \psi _\alpha \Vert _2 \ge \Vert \psi _\alpha \Vert _2 + R^{-1} \Vert x ( \nabla \chi ^{R,z} ( w) ) \psi _\alpha \Vert _2$ for $w \in B_R (z)$ and thus with the ground state’s scaling properties (3.15) we have $\Vert \chi ^{R,z} \psi _\alpha \Vert _2 \ge C$ for sufficiently large $\alpha \ge \alpha _0$. Hence we find with (3.19), (3.38) and the scaling of $\chi ^{R,z}$ at $\Vert \Delta \psi ^{R,z}_\alpha \Vert _2 \le C \sqrt{\alpha }$ and thus

3.66 $$\begin{aligned} \vert \widehat{\varrho }_{\psi _\alpha ^{R,z}} (k) \vert \le \frac{C \alpha ^{1/2}}{\vert k \vert ^2} \; . \end{aligned}$$

From (3.64) and (3.66) we find that for high momenta, i.e. all $k \in B_{R_2}^c = \lbrace k \in \mathbb {R}^3: \vert k \vert > R_2 \rbrace$ we have

3.67 $$\begin{aligned} \Vert \widehat{\varrho }_{\psi _\alpha } - \widehat{\varrho }_{\psi ^{R,z}_\alpha } \Vert _{L^2( B_{R_2}^c )} \le \frac{C\alpha ^{1/2}}{R_2^{1/2}} \; . \end{aligned}$$

We remark that we choose $R_2:= R_2 ( \alpha ) = \alpha ^{1/5}$, however, for simplicity (as for *R*), we neglect the dependence of $\alpha$ in its notation. For low momenta, i.e. $k \in B_{R_2}$ we use that by Assumption (1.1) we have $\vert \widehat{g} (k) \vert \ge ( 1 + \vert k \vert )^{-9/2}$ and thus

3.68 $$\begin{aligned} \Vert \widehat{\varrho }_{\psi _\alpha } - \widehat{\varrho }_{\psi ^{R,z}_\alpha } \Vert _{L^2( B_{R_2})} \le&C ( 1 + \vert R_2 \vert )^{9/2} \Vert v \ \varepsilon ^{-1/2} ( \widehat{\varrho }_{\psi _\alpha } - \widehat{\varrho }_{\psi ^{R,z}_\alpha } ) \Vert _{L^2( B_{R_2})} \nonumber \\ \le&C ( 1 + \vert R_2 \vert )^{9/2} \Vert v \ \varepsilon ^{-1/2} ( \widehat{\varrho }_{\psi _\alpha } - \widehat{\varrho }_{\psi ^{R,z}_\alpha } ) \Vert _{2} \; \end{aligned}$$

and we find with (3.27) (assuming $R_2 \le \alpha ^{1/5}$)

3.69 $$\begin{aligned} \Vert \widehat{\varrho }_{\psi _\alpha } - \widehat{\varrho }_{\psi ^{R,z}_\alpha } \Vert _2 \le&C \alpha ^{-1/3} ( 1 + \vert R_2 \vert )^{9/2} \; . \end{aligned}$$

Optimizing with respect to $R_2$ leads to $R_2 = \alpha ^{1/5}$ and thus for sufficiently large $\alpha \ge \alpha _0$ to

3.70 $$\begin{aligned} \Vert \widehat{\varrho }_{\psi _\alpha } - \widehat{\varrho }_{\psi ^{R,z}_\alpha } \Vert _2 \le&C \alpha ^{2/5} \; . \end{aligned}$$

In particular it follows as $\psi _\alpha ^{R,z}$ and $\psi _\alpha$ have the same phase

3.71 $$\begin{aligned} \Vert \psi _\alpha - \psi _\alpha ^{R,z} \Vert _4^4 =\Vert \vert \psi _\alpha \vert -\vert \psi _\alpha ^{R,z} \vert \Vert _4^4 \le \Vert \widehat{\varrho }_{\psi _\alpha } - \widehat{\varrho }_{\psi ^{R,z}_\alpha } \Vert _2^2 \le C \alpha ^{4/5} . \end{aligned}$$

We recall that we need to estimate the $L^2$-norm difference of $\psi _\alpha , \psi _\alpha ^{R,z}$ on the ball $B_{R_1}$, i.e. we have by Cauchy Schwarz’s inequality with $R_1= \alpha ^{-1/5}$

3.72 $$\begin{aligned} \Vert \psi _\alpha - \psi _\alpha ^{R,z} \Vert _{L^2 ( B_{R_1})}^2 \le C R_1^{3/2} \alpha ^{2/5} \le C \alpha ^{-1/5} \; \end{aligned}$$

and we arrive with (3.61) at

3.73 $$\begin{aligned} \inf _\theta \Vert e^{i \theta } \psi _\alpha - \psi _\textrm{osc}\Vert _{2}^2 \le C \alpha ^{-1/10} \; . \end{aligned}$$

**Proof of part** *(c)***:** In order to prove $H^1$-norm convergence of $\psi _\alpha$ to $\psi _\textrm{osc}$ we observe that from the Euler-Lagrange equations of $\psi _\alpha$ resp. $\psi _\textrm{osc}$, Assumption 1.1 and the scaling properties of $\psi _\textrm{osc}$

3.74 $$\begin{aligned} - \tfrac{1}{2m} \Delta \left( \psi _\alpha - \psi _\textrm{osc}\right) =&\left( \alpha \left( h * \vert \psi _\alpha \vert ^2 \right) + \mu _{\psi _\alpha } \right) \psi _\alpha - \left( \alpha \left( h * \vert \psi _\textrm{osc}\vert ^2 \right) + \mu _{\psi _\textrm{osc}} \right) \psi _\textrm{osc}\nonumber \\&+ C \left( \alpha ( \vert \cdot \vert ^4 * \vert \psi _\textrm{osc}\vert ^2 ) + 1 \right) \psi _\textrm{osc}\end{aligned}$$

for a constant $C>0$ independent of $\alpha$. We recall that both $\psi _\alpha$ and $\psi _\textrm{osc}$ are $L^2$-normalized functions. Hence introducing the notation $\widetilde{h} = h - \Vert g \Vert _2^2$ and

3.75 $$\begin{aligned} \widetilde{\mu }_\psi = \langle \psi |-\tfrac{\Delta }{2m} - \alpha ( \widetilde{h} * \vert \psi \vert ^2 )|\psi \rangle \end{aligned}$$

for any $\psi \in H^1 ( \mathbb {R}^3)$ we arrive at

3.76 $$\begin{aligned}&- \tfrac{1}{2m} \Delta \left( \psi _\alpha - \psi _\textrm{osc}\right) = \alpha \left( \widetilde{h} * \psi _\alpha \overline{\left( \psi _\alpha - \psi _\textrm{osc}\right) } \right) \psi _\alpha + \alpha \left( \widetilde{h} * \left( \psi _\alpha - \psi _\textrm{osc}\right) \overline{ \psi _\textrm{osc}} \right) \psi _\alpha \nonumber \\&\quad + \alpha \left( \widetilde{h} *\vert \psi _\textrm{osc}\vert ^2 \right) \left( \psi _\textrm{osc}- \psi _\alpha \right) - \left( \widetilde{\mu }_{\psi _\alpha } - \widetilde{\mu }_{\psi _\textrm{osc}} \right) \psi _\alpha + \widetilde{\mu }_{\psi _\alpha } \left( \psi _\textrm{osc}- \psi _\alpha \right) \nonumber \\&\quad + \widetilde{\mu }_{\psi _\textrm{osc}} \left( \psi _\alpha - \psi _\textrm{osc}\right) + C \alpha ( \vert \cdot \vert ^4 * \vert \psi _\textrm{osc}\vert ^2 ) \psi _\textrm{osc}\end{aligned}$$

From Assumption 1.1 we have $\vert \widetilde{h} (x) \vert \le C x^2$ and thus together with the ground state’s scaling properties (part *(c)*) we find $\vert \widetilde{\mu }_{\psi _\alpha } \vert \le C \alpha ^{1/2}$, $\vert \widetilde{\mu }_{\psi _\textrm{osc}} \vert \le C \alpha ^{1/2}$ and

3.77 $$\begin{aligned} \vert \widetilde{\mu }_{\psi _\alpha } - \widetilde{\mu }_{\psi _\textrm{osc}} \vert = \vert \mu _{\psi _\alpha } - \mu _{\psi _\textrm{osc}} \vert \le C \alpha ^{5/12} \end{aligned}$$

from part (part *(a)*). Therefore we find with the approximation of the ground state (part *(b)*) and the ground state’s scaling properties (part *(c)*) that

3.78 $$\begin{aligned} \vert \left\langle \psi _\textrm{osc}\right| \left| - \Delta \left( \psi _\alpha - \psi _\textrm{osc}\right) \right\rangle \vert , \vert \left\langle \psi _\alpha \right| \left| - \Delta \left( \psi _\alpha - \psi _\textrm{osc}\right) \right\rangle \vert \le C \alpha ^{9/20} \end{aligned}$$

finally yielding

3.79 $$\begin{aligned} \Vert \nabla \psi _\alpha - \nabla \psi _\textrm{osc}\Vert _2^2 \le C \alpha ^{9/40} \end{aligned}$$

and the desired lower and upper bound in (3.19).

**Proof of** *(d)***:** To prove convergence of $\psi _\alpha$ to $\psi _\textrm{osc}$ for $\psi _\alpha$ satisfying (3.14) in the weighted $L^2$-norm, too, we proceed similarly as in part *(d)*. From the Euler-Lagrange equation of $\psi _\alpha$ we get

3.80 $$\begin{aligned}&\left( \tfrac{1}{2m} \Delta - \mu _{\psi _\alpha } \right) \left( \psi _\alpha - \psi _\textrm{osc}\right) \nonumber \\&\quad =2 \alpha \left( h * \vert \psi _\alpha \vert ^2 \right) \psi _\alpha - 2\alpha \left( \left( h * \vert \psi _\textrm{osc}\vert ^2 \right) + ( \mu _{\psi _\textrm{osc}} - \mu _{\psi _\alpha }) \right) \psi _\textrm{osc}\nonumber \\&\quad = 2 \alpha \left( h * \psi _\alpha \overline{\left( \psi _\alpha - \psi _\textrm{osc}\right) } \right) \psi _\alpha +2 \alpha \left( h * \left( \psi _\alpha - \psi _\textrm{osc}\right) \overline{ \psi _\textrm{osc}} \right) \psi _\alpha \nonumber \\&\quad \quad + 2 \alpha \left( h *\vert \psi _\textrm{osc}\vert ^2 \right) \left( \psi _\textrm{osc}- \psi _\alpha \right) - \left( \mu _{\psi _\alpha } - \mu _{\psi _\textrm{osc}} \right) \psi _\textrm{osc}+ C \alpha ( \vert \cdot \vert ^4 * \vert \psi _\textrm{osc}\vert ^2 ) \psi _\textrm{osc}\; . \end{aligned}$$

By assumption resp. part *(a)* we have

3.81 $$\begin{aligned} {{\,\textrm{dist}\,}}_{L^2} \left( \psi _\textrm{osc}, \Theta (\psi _\alpha ) \right) = \Vert \psi _\alpha - \psi _\textrm{osc}\Vert _2 \le C \alpha ^{-1/20} \end{aligned}$$

and furthermore we have from (3.58) (using assumption (3.14)) the estimate

3.82 $$\begin{aligned} \Vert x^2 (\psi _\alpha - \psi _\textrm{osc}) \Vert _2 \le C \alpha ^{-1/2} \Vert F \Vert _2 \; . \end{aligned}$$

where *F* denotes the r.h.s. of (3.80). It follows from the approximation of the Lagrange multiplier (part *(a)*) and the ground state (part *(b)*) that $\Vert F \Vert _2 \le C \alpha ^{-1/20}$ and we finally arrive at the desired bound of part *(c)*. $\square$

### Properties of the Hessian

The Hessian $\mathcal {H}_{\alpha }$ of $\mathcal {E}_\alpha$ is defined for any $\psi _\alpha \in \mathcal {M}_{\mathcal {E}_\alpha }$ by

3.83 $$\begin{aligned} \mathcal {H}_{\alpha }:= \lim _{\varepsilon \rightarrow 0} \frac{1}{\delta ^2} \left( \mathcal {E}_\alpha \left( \frac{\psi _\alpha + \delta f}{ \Vert \psi _\alpha + \delta f \Vert _2} \right) - e_\alpha \right) \quad \text {for all} \quad f \in H^1 ( \mathbb {R}^3 ) \; . \end{aligned}$$

We can explicitly compute the Hessian and find

3.84 $$\begin{aligned} \mathcal {H}_\alpha := \langle \mathrm{Im\,}f|\textrm{H}_{\alpha }|\mathrm{Im\,}f\rangle + \langle \mathrm{Re\,}f|Q_\alpha \left( {\textrm{H}}_\alpha - 4 X_{\psi _\alpha }\right) Q_\alpha |\mathrm{Re\,}f\rangle \end{aligned}$$

where $$Q_\alpha = 1 - P_\alpha = 1 - \left| \psi _\alpha \right\rangle \left\langle \psi _\alpha \right| $$ and

3.85 $$\begin{aligned} {\textrm{H}}_\alpha = {\textrm{h}}_{\sqrt{\alpha } \varphi _\alpha } - \mu _{\psi _\alpha }, \quad \text {and} \quad X_{\psi _\alpha } (x;y) = \psi _\alpha (x) \; h(x-y) \; \psi _\alpha (y) \; . \end{aligned}$$

#### Positivity of the Hessian

We compare the Hessian’s components

3.86 $$\begin{aligned} \mathcal {H}_\alpha ^{(1)}&:= \inf _{\psi _\alpha \in \mathcal {M}_{\mathcal {E}_\alpha }} \inf _{\begin{array}{c} f \in H^1( \mathbb {R}^3), \Vert f \Vert _2 =1\\ f \in \left( \textrm{span} \lbrace \psi _\alpha \rbrace \right) ^\perp \end{array} } \langle f|{\textrm{H}}_\alpha |f\rangle , \nonumber \\ \mathcal {H}_\alpha ^{(2)}&:= \inf _{\psi _\alpha \in \mathcal {M}_{\mathcal {E}_\alpha }} \inf _{\begin{array}{c} f \in H^1( \mathbb {R}^3), \Vert f \Vert _2 =1\\ f \in \left( \textrm{span} \lbrace \psi _\alpha , \partial _1 \psi _\alpha , \partial _2 \psi _\alpha , \partial _3 \psi _\alpha \rbrace \right) ^\perp \end{array} } \langle f|{\textrm{H}}_\alpha - 4X_{\psi _\alpha }|f\rangle \end{aligned}$$

with $\mathcal {H}_\textrm{osc}$ (defined in (3.10)) known to satisfy $\mathcal {H}_\textrm{osc} \ge C \sqrt{\alpha }$ for a constant $C>0$ independent of $\alpha$. We remark that by definition the Hessian $\mathcal {H}_\alpha$ is defined modulo its zero modes namely the ground state $\psi _\alpha$ for the first component resp. $\psi _\alpha$ and its partial derivatives $\partial _j \psi _\alpha$ for $i=1,2,3$ for the second component.

##### Lemma 3.3

Let $\varepsilon , v$ satisfy Assumption 1.1. Then, there exists $\alpha _0>0$ and $C>0$ (independent of $\alpha$) such that

3.87 $$\begin{aligned} \mathcal {H}_\alpha ^{(i)} \ge C \alpha ^{1/2}, \quad \text {for all} \quad \alpha \ge \alpha _0 \; . \end{aligned}$$

##### Proof

We present the proof of the Hessian’s component $\mathcal {H}_\alpha ^{(2)}$. The statement for $\mathcal {H}^{(1)}$ then follows with similar arguments.

For any $\psi _\alpha \in \mathcal {M}_{\mathcal {E}_\alpha }$ and $f \in H^1 ( \mathbb {R}^3)$ we define the projection $Q_\alpha ' = 1- P_\alpha ', P_\alpha ' = \left| \psi _\alpha \right\rangle \left\langle \psi _\alpha \right| + \sum _{j=1}^3 \left| \partial _j \psi _\alpha \right\rangle \left\langle \partial _j \psi _\alpha \right| / \Vert \partial _j \psi _\alpha \Vert _2^2$ and furthermore the $L^2$-normalized function

3.88 $$\begin{aligned} g_\alpha := \frac{Q_\alpha ' f}{\Vert Q_\alpha ' f \Vert _2} \; . \end{aligned}$$

Thus in the following we consider the expectation value

3.89 $$\begin{aligned} \langle g_\alpha |{\textrm{H}}_\alpha - 4X_{\psi _\alpha }|g_\alpha \rangle = \langle g_\alpha |{\textrm{H}}_\alpha -4 \widetilde{X}_{\psi _\alpha } | g_\alpha \rangle \end{aligned}$$

where we introduced the notation

3.90 $$\begin{aligned} \widetilde{X}_{\psi _\alpha } (x;y)= \psi _\alpha (x) \; \widetilde{h} (x-y) \; \psi _\alpha (y) \; \end{aligned}$$

with $\widetilde{h} = h - \Vert g \Vert _2^2$ and used that the zero-th order term of the expansion of $h$ in the above expectation value vanishes as $Q'_\alpha f$ is orthogonal to $\psi _\alpha$. By translational invariance of the problem we restrict to $\psi _\alpha$ such that ${{\,\textrm{dist}\,}}_{L^2} ( \Theta (\psi _\alpha ), \psi _\textrm{osc}) = \Vert \psi _\textrm{osc}- \psi _\alpha \Vert _2$ (so that Lemma 3.2*(c), (d)* apply). We recall that we want to compare $\mathcal {H}_\alpha ^{(i)}$ with $\mathcal {H}_\textrm{osc}$ that is of order $\sqrt{\alpha }$. Thus any term we can show to be $o( \sqrt{\alpha })$ will be dominated in the end by $\mathcal {H}_\textrm{osc}$ and thus will be considered to be sub-leading for sufficiently large $\alpha \ge \alpha _0$.

We shall first show that $g_\alpha$ is approximately orthogonal to the harmonic oscillator’s ground and first excited state $P_\textrm{osc}' = \left| \psi _\textrm{osc}\right\rangle \left\langle \psi _\textrm{osc}\right| + \sum _{j=1}^3 \left| \partial _j \psi _\textrm{osc}\right\rangle \left\langle \partial _j \psi _\textrm{osc}\right| / \Vert \partial _j \psi _\textrm{osc} \Vert _2^2$, i.e. that it is enough to consider

3.91 $$\begin{aligned} \langle Q_\textrm{osc}' g_\alpha | {\textrm{H}}_\alpha -4 \widetilde{X}_{\psi _\alpha } | Q_\textrm{osc}' g_\alpha \rangle \; . \end{aligned}$$

This follows from the observation that the difference is given by

3.92 $$\begin{aligned}&\langle g_\alpha |{\textrm{H}}_\alpha - 4\widetilde{X}_{\psi _\alpha } | g_\alpha \rangle - \langle Q_\textrm{osc}' g_\alpha | {\textrm{H}}_\alpha - 4\widetilde{X}_{\psi _\alpha } | Q_\textrm{osc}' g_\alpha \rangle \nonumber \\&\quad = 2 \mathrm{Re\,}\left\langle P_\textrm{osc}'g_\alpha \right| {\textrm{H}}_\alpha -4\widetilde{X}_{\psi _\alpha } \left| g_\alpha \right\rangle + \left\langle P_\textrm{osc}'g_\alpha \right| {\textrm{H}}_\alpha -4\widetilde{X}_{\psi _\alpha } \left| P_\textrm{osc}' g_\alpha \right\rangle \; . \end{aligned}$$

On the one hand, since

3.93 $$\begin{aligned} \Vert P_\textrm{osc} Q_\alpha g_\alpha \Vert _2 \le C \Vert \psi _\alpha - \psi _\textrm{osc}\Vert _2 \Vert g_\alpha \Vert _2 \le C \alpha ^{-1/20} \Vert g_\alpha \Vert _2 \end{aligned}$$

and similarly denoting $P_\textrm{osc}^{(j)} =: \left| \partial _j \psi _\textrm{osc}\right\rangle \left\langle \partial _j \psi _\textrm{osc}\right| / \Vert \partial _j \psi _\textrm{osc} \Vert _2^2$ and $P_{\alpha }^{(j)} =: \left| \partial _j \psi _\alpha \right\rangle \left\langle \partial _j \psi _\alpha \right| / \Vert \partial _j \psi _\alpha \Vert _2^2$,

3.94 $$\begin{aligned} \Vert P_\textrm{osc}^{(j)} Q_\alpha ^{(j)} g_\alpha \Vert _2 \le C \Vert \psi _\textrm{osc}\Vert _2^{-1} \Vert \partial _j \psi _\textrm{osc}- \psi _\alpha \Vert _2 \Vert g_\alpha \Vert _2 \le C \alpha ^{-1/40} \end{aligned}$$

from Lemma 3.2*(d)* for sufficiently large $\alpha \ge \alpha _0$ and $\Vert \nabla \psi _\textrm{osc}\Vert \ge c \alpha ^{1/4}$ for some $c>0$. Thus we arrive at

3.95 $$\begin{aligned} \Vert P'_\textrm{osc} g_\alpha \Vert _2 \le C \alpha ^{-1/40}. \end{aligned}$$

On the other hand we have

3.96 $$\begin{aligned} {\textrm{H}} = \textrm{h}_{\sqrt{\alpha } \varphi _\textrm{osc}} - \mu _{\psi _\textrm{osc}} + ( \mu _{\psi _\alpha } - \mu _{\psi _\textrm{osc}}) \end{aligned}$$

so that with Lemma 3.2*(d)* we have $\Vert \textrm{H}_{\alpha } P'_\textrm{osc} f \Vert _2 \le C \alpha ^{1/2}$ and we arrive at

3.97 $$\begin{aligned} \langle g_\alpha |{\textrm{H}}_\alpha - 4\widetilde{X}_{\psi _\alpha } | g_\alpha \rangle - \langle Q_\textrm{osc}' g_\alpha | {\textrm{H}}_\alpha - 4\widetilde{X}_{\psi _\alpha } | Q_\textrm{osc}' g_\alpha \rangle \ge - C \alpha ^{-19/40} \; . \end{aligned}$$

As a next step, we shall replace $Q_\textrm{osc} g_\alpha$ with the $L^2$-normalized function

3.98 $$\begin{aligned} g_\textrm{osc} := \frac{Q'_\textrm{osc} Q_\alpha ' f }{ \Vert Q'_\textrm{osc} Q_\alpha ' f\Vert _2} \; \end{aligned}$$

For this we first observe that

3.99 $$\begin{aligned} Q'_\alpha g_\alpha = \frac{ \Vert Q'_\textrm{osc} Q_\alpha ' f\Vert _2 }{ \Vert Q_\alpha ' f\Vert _2} g_\textrm{osc} \end{aligned}$$

and thus, we need to control the normalization constants’ ratio. For this we use (3.95) and find that

3.100 $$\begin{aligned} \Vert Q_\alpha ' f \Vert _2^2 =&\Vert Q_\textrm{osc}' Q_\alpha ' f\Vert _2^2 + \Vert P'_\textrm{osc} Q_\alpha ' f \Vert _2^2 \le \Vert Q_\textrm{osc}' Q_\alpha ' f\Vert _2^2 + C \alpha ^{-1/12} \Vert Q_\alpha ' f \Vert _2 \end{aligned}$$

for a constant $C>0$ independent in $\alpha$. In particular we obtain

3.101 $$\begin{aligned} \Vert Q_\alpha ' f \Vert _2 \ge C (1- \alpha ^{-1/40} )^{-1} \Vert Q_\textrm{osc}' Q_\alpha ' f\Vert _2 \; \end{aligned}$$

for sufficiently large $\alpha \ge \alpha _0$. Thus from (3.97) and (3.98) we get

3.102 $$\begin{aligned} \langle g_\alpha |{\textrm{H}}_\alpha - 4\widetilde{X}_{\psi _\alpha } | g_\alpha \rangle \ge C (1- \alpha ^{-1/40} )^{-2} \langle g_\textrm{osc} | {\textrm{H}}_\alpha -4 \widetilde{X}_{\psi _\alpha } | g_\textrm{osc} \rangle - C \alpha ^{19/40} \; . \end{aligned}$$

Next we show that the operator $\widetilde{X}_{\psi _\alpha }$ contributes sub-leading (i.e. $o ( \sqrt{\alpha })$) only. For this we write

3.103 $$\begin{aligned} \widetilde{X}_{\psi _\alpha } - \widetilde{X}_{\psi _\textrm{osc}} = \left( \psi _\alpha (x) - \psi _\textrm{osc}(x) \right) \widetilde{h}( x-y) \psi _\alpha (y) + \psi _\textrm{osc}(x) \widetilde{h} (x-y) \left( \psi _\alpha (y) - \psi _\textrm{osc}(y) \right) \; . \end{aligned}$$

With $\vert \widetilde{h} (x) \vert \le C x^2$ we find from Lemma 3.2*(c)* and $\Vert g_\textrm{osc} \Vert _2 =1$ that

3.104 $$\begin{aligned} \langle g_\textrm{osc}|\widetilde{X}_{\psi _\alpha }|g_\textrm{osc}\rangle \ge \langle g_\textrm{osc}|\widetilde{X}_{\psi _\textrm{osc}}|g_\textrm{osc}\rangle - C \alpha ^{9/20} \; . \end{aligned}$$

We recall that $g_\textrm{osc}$ is orthogonal to $\psi _\textrm{osc}$ and its partial derivatives. In particular, as $\nabla \psi _\textrm{osc}= x \psi _\textrm{osc}$, the function $g_\textrm{osc}$ is orthogonal to $x \psi _\textrm{osc}$, too. Therefore not only the zero-th but also the first-order term of the Taylor expansion of *h* in $\widetilde{X}_{\psi _\textrm{osc}}$ evaluated in $g_\textrm{osc}$ vanishes, i.e.

3.105 $$\begin{aligned} \langle g_\textrm{osc}|\widetilde{X}_{\psi _\textrm{osc}}|g_\textrm{osc}\rangle = \langle g_\textrm{osc}|\widetilde{\widetilde{X}}_{\psi _\textrm{osc}}|g_\textrm{osc}\rangle \end{aligned}$$

where $\widetilde{\widetilde{X}}_{\psi _\textrm{osc}}(x,y) = \alpha \psi _\textrm{osc}(x) ( h (x-y) - \Vert g \Vert _2^2 -\Vert \nabla g \Vert _2^2 (x-y)^2 ) \psi _\textrm{osc}(y)$. Since $\vert \widetilde{h}(x) -\Vert \nabla g \Vert _2^2 x^2\vert \le C x^4$ by Assumption 1.1 we find with the harmonic oscillators scaling properties that $\langle g_\textrm{osc}|\widetilde{\widetilde{X}}_{\psi _\textrm{osc}}|g_\textrm{osc}\rangle \ge - C$, and thus from (3.102)

3.106 $$\begin{aligned} \langle g_\alpha | {\textrm{H}}_\alpha - 4\widetilde{X}_{\psi _\alpha }|g_\alpha \rangle \ge C (1- \alpha ^{-1/40} )^{-2} \langle g_\textrm{osc}|{\textrm{H}}_\alpha |g_\textrm{osc}\rangle - C \alpha ^{19/40} \end{aligned}$$

for sufficiently large $\alpha \ge \alpha _0$. Now it remains to compare the r.h.s. with the harmonic oscillator. For this we split the operator ${\textrm{H}}_\alpha$ into one part that is localized on a ball $B_R$ with $R= \alpha ^{-1/6}$ (that we shall show is bounded from below by $\mathcal {H}_\textrm{osc}$ that is $O( \sqrt{\alpha })$) and a part outside $B_R^c$ (that we will show is bounded from below by a positive constant of $O( \alpha )$, i.e. trivially satisfying the claim for sufficiently large $\alpha \ge \alpha _0$).

For the localization we consider a partition of unity $0 \le \eta ^1, \eta ^2 \le 1$ with $\eta ^i \in C_0^\infty ( \mathbb {R}^3)$ and

3.107 $$\begin{aligned} \eta ^1 (x) = {\left\{ \begin{array}{ll} 1 &  x \in B_1 \\ 0 &  x \in B_2^c \\ \end{array}\right. }, \quad \eta ^2 = \sqrt{1- \vert \eta ^1 \vert ^2} \; . \end{aligned}$$

and define the rescaled version $\eta _R^i (x) = \eta ^i (x/R )$ and the $L^2$-normalized function

3.108 $$\begin{aligned} g^i_R = \eta _R^i g_\textrm{osc} / \Vert \eta _R^i g_\textrm{osc} \Vert _2 \; . \end{aligned}$$

With standard arguments of IMS localization we find

3.109 $$\begin{aligned} \langle g_\textrm{osc}|{ \mathrm H}_\alpha |g_\textrm{osc}\rangle =&\sum _{i=1}^2 \Vert \eta _R^i g_\textrm{osc} \Vert _2^2 \langle g_R^i|{\textrm{H}}_\alpha |g_R^i\rangle - \sum _{i=1}^2 \langle g_\textrm{osc}|\vert \nabla \eta _R^i\vert ^2| g_\textrm{osc}\rangle \; . \end{aligned}$$

By scaling the last summand is of order $R^{-2} = \alpha ^{1/3}$ yielding

3.110 $$\begin{aligned} \langle g_\textrm{osc}|{ \mathrm H}_\alpha |g_\textrm{osc}\rangle \ge&\sum _{i=1}^2 \Vert \eta _R^i g \Vert _2^2 \langle g_R^i|{\textrm{H}}_\alpha |g_R^i\rangle - C\alpha ^{1/3} \end{aligned}$$

and it remains to estimate the expectation value $\langle f_R^i|{\textrm{H}}_\alpha |f_R^i\rangle$ for $i=1,2$.

We start with the expectation value w.r.t $g_{R}^2$ supported on $B_R^c$. Since $\cos (k \cdot x ) \le 1$, we find

3.111 $$\begin{aligned} \langle g_R^2|{ \mathrm H}_\alpha |g_R^2\rangle \ge&- \mu _{\psi _\alpha }- \langle g_R^2|\sqrt{\alpha } V_{\sqrt{\alpha }\varphi _\alpha }|g_R^2\rangle \nonumber \\ \ge&2 \alpha \Vert g \Vert _2^2 + \langle g_R^2|\sqrt{\alpha } V_{\sqrt{\alpha }\varphi _\alpha }|g_R^2\rangle \; . \end{aligned}$$

To show that the remaining term contributes sub-leading (i.e. $o(\alpha )$) only, we need to control the $L^\infty$- norm of $V_{\sqrt{\alpha }\varphi _\alpha }$ on $B_R^{c}$, i.e.

3.112 $$\begin{aligned} \sqrt{\alpha }\vert V_{\sqrt{\alpha }\varphi _\alpha } (x) \vert \le C \alpha \int \vert h( x-y )\vert \vert \psi _{\alpha } (y)\vert ^2 dy + C \alpha ^{5/12} \; \end{aligned}$$

for $\vert x \vert \ge \alpha ^{-1/6}$. We split the integral in $B_{\widetilde{R}}$ and $B_{\widetilde{R}}^c$ where now we choose $\widetilde{R} = \alpha ^{-1/5}$. For $y \in B_{\widetilde{R}}^c$ we find by the scaling properties of $\psi _\alpha$ that

3.113 $$\begin{aligned} \Vert \psi _{\alpha } \Vert _{L^2( B_{\widetilde{R}}^c)}^2 \le C \alpha ^{2/5} \Vert x \psi _{\alpha } \Vert _{L^2( B_{\widetilde{R}}^c)}^2 \le \alpha ^{-1/10} \end{aligned}$$

and we arrive with $\Vert h \Vert _{L^\infty ( \mathbb {R}^3)}\le C$ for $\vert x \vert \ge \alpha ^{-1/6}$ at

3.114 $$\begin{aligned} \sqrt{\alpha }\vert V_{\sqrt{\alpha }\varphi _\alpha } (x) \vert \le C \alpha \int _{\vert y \vert \le \alpha ^{-1/5}} \vert h( x-y )\vert \vert \psi _{\alpha } (y)\vert ^2 dy + C \alpha ^{9/10} \; . \end{aligned}$$

Now let $\vert y\vert \le \alpha ^{-1/5}$ and $\vert x \vert \ge \alpha ^{-1/6}$. Then we have $\vert x - y \vert \ge \vert x \vert - \vert y \vert \ge C \alpha ^{-1/6}$ for sufficiently large $\alpha \ge \alpha _0$. Thus, with $\Vert h \Vert _{L^\infty ( \mathbb {R}^3)} \le C$

3.115 $$\begin{aligned} \sqrt{\alpha }\vert V_{\sqrt{\alpha }\varphi _\alpha } (x) \vert \le C \alpha ^{1+1/6} \int _{\vert y \vert \le \alpha ^{-1/5}} \vert x- y \vert \vert \psi _{\alpha } (y)\vert ^2 dy + C \alpha ^{5/12} \end{aligned}$$

for $\vert x \vert \ge \alpha ^{-1/6}$. With Cauchy Schwarz inequality and $\Vert \psi _{\alpha } \Vert _4 \le C \Vert \psi _{\alpha } \Vert _{H^1} \le C \alpha ^{1/4}$ (from Lemma 3.2*(d)*) we find

3.116 $$\begin{aligned} \sqrt{\alpha }\vert V_{\sqrt{\alpha }\varphi _\alpha } (x) \vert \le C \alpha ^{1+1/6 + 1/4-1/2} + C \alpha ^{5/12} \le C \alpha ^{11/12} \; . \end{aligned}$$

Hence we deduce from (3.111)

3.117 $$\begin{aligned} \langle g_R^2|{ \mathrm H}_\alpha |g_R^2\rangle \ge 2 \alpha \Vert g \Vert _2^2 - C_2 \alpha ^{11/12} \ge C_1 \alpha \end{aligned}$$

for constant $C_1,C_2$ independent of $\alpha$ and sufficiently large $\alpha \ge \alpha _0$.

We recall that the goal for the expectation value

3.118 $$\begin{aligned} \langle g_R^1|{ \mathrm H}_\alpha |g_R^1\rangle \end{aligned}$$

with $f_R^1$ supported on $B_R$ is a comparison with the Hessian of the harmonic oscillator $\mathcal {H}_\textrm{osc}$ that is $O( \sqrt{\alpha })$. For this we observe that $f_R^1$ is almost orthogonal to $\psi _\textrm{osc}$ and its partial derivatives as

3.119 $$\begin{aligned} \Vert P_\textrm{osc} g_R^1 \Vert _2 \le C \Vert \psi _\textrm{osc} \Vert _{L^2( B_R^c)} \le C \alpha ^{-1/4} \end{aligned}$$

by Lemma 3.2*(c)*) and similarly for the partial derivatives. Thus (with similar arguments as in the beginning of this proof (see Eq. (3.91) and subsequent)) instead of $g_R^1$ we consider in the following the $L^2$-normalized function

3.120 $$\begin{aligned} \widetilde{g}_R^1 := \frac{Q_\textrm{osc}' g_R^1}{\Vert Q_\textrm{osc}' g_R^1 \Vert _2 } \; \end{aligned}$$

paying a price sub-leading in $\alpha$ (i.e. $o( \sqrt{\alpha })$ and given by

3.121 $$\begin{aligned} \left\langle g_R^1\right| {\textrm{H}}_\alpha \left| f_R^1\right\rangle \ge (1- \alpha ^{-1/12})^2 \left\langle \widetilde{g}_R^1\right| {{ \mathrm H}}_\alpha \left| \widetilde{g}_R^1\right\rangle - C \alpha ^{1/3} \; . \end{aligned}$$

We use the Taylor expansion of $h$, $\langle \psi _\alpha |x|\psi _\alpha \rangle =0$ and Lemma 3.2*(d)* and find

3.122 $$\begin{aligned} \langle \widetilde{g}_R^1|{ \mathrm H}_\alpha |\widetilde{g}_R^1\rangle = \langle \widetilde{g}_R^1| \textrm{h}_{\sqrt{\alpha }\varphi _\alpha } - \mu _{\psi _\alpha }|\widetilde{g}_R^1\rangle \ge \langle \widetilde{f}_R^1| \textrm{h}_\textrm{osc} - \left\langle \psi _\alpha \right| \textrm{h}_\textrm{osc}\left| \psi _\alpha \right\rangle |\widetilde{f}_R^1\rangle - C \alpha ^{1/3} \; \end{aligned}$$

and thus (since $\left\langle \psi _\alpha \right| \textrm{h}_\textrm{osc}\left| \psi _\alpha \right\rangle \ge \left\langle \psi _\textrm{osc}\right| \textrm{h}_\textrm{osc}\left| \psi _\textrm{osc}\right\rangle$)

3.123 $$\begin{aligned} \langle \widetilde{g}_R^1|{ \mathrm H}_\alpha |\widetilde{g}_R^1\rangle \ge \langle \widetilde{g}_R^1|{ \mathrm H}_\textrm{osc} |\widetilde{g}_R^1\rangle - C \alpha ^{1/3} \; . \end{aligned}$$

By construction $\widetilde{g}_R^1$ is a $L^2$-normalized function and orthogonal to the harmonic oscillator’s ground state and its partial derivatives. Thus $\widetilde{g}_R^1$ is a competitor for a minimizer of the harmonic oscillator’s Hessian and we conclude that

3.124 $$\begin{aligned} \langle g_R^1|{ \mathrm H}_\alpha |g_R^1\rangle \ge (1- \alpha ^{-1/40})^2 \mathcal {H}_\textrm{osc} \; - C \alpha ^{19/40} \; . \end{aligned}$$

Since $\mathcal {H}^{(2)}$ is a convex combination of (3.117) and (3.124), we find

3.125 $$\begin{aligned} \mathcal {H}^{(2)} \ge C (1- \alpha ^{-1/40})^4 \mathcal {H}_\textrm{osc} - C \alpha ^{19/40} \end{aligned}$$

and conclude that there exists $\alpha \ge \alpha _0$ such that $\mathcal {H}^{(2)} \ge C \alpha ^{1/2}$ for all $\alpha \ge \alpha _0$. $\square$

The Hessian’s positivity in the strong coupling limit $\alpha \rightarrow \infty$ leads to local coercivity estimates summarized in the following Corollary.

##### Corollary 3.1

There exists $\alpha _0 >0$ and $\kappa , C >0$ (independent of $\alpha$) such that for all $\alpha > \alpha _0$ and $\psi _\alpha \in \mathcal {M}_{\mathcal {E}_\alpha }$, any $L^2$-normalized $\psi \in H^1 ( \mathbb {R}^3)$ and $\varphi \in L^2 ( \mathbb {R}^3)$ with

3.126 $$\begin{aligned} {{\,\textrm{dist}\,}}_{L^2} \left( \Theta ( \psi _\alpha ), \; \psi \right) \le \kappa \alpha ^{-1/2} \end{aligned}$$

we have

3.127 $$\begin{aligned} \mathcal {G}_\alpha ( \psi , \varphi ) - e_\alpha&\ge C\sqrt{\alpha } {{\,\textrm{dist}\,}}_{L^2} \left( \Theta (\psi _\alpha ) , \; \psi \right) ^2 \; ,\end{aligned}$$

3.128 $$\begin{aligned} \mathcal {G}_\alpha (\psi , \varphi ) - e_\alpha&\ge \frac{C}{\sqrt{\alpha }} {{\,\textrm{dist}\,}}_{L^2_{\sqrt{\varepsilon }}} \left( \Omega ( \varphi _\alpha ), \; \varphi \right) ^2 \; . \end{aligned}$$

The proof is based on an expansion of $\mathcal {G}_\alpha$ around the ground state energy $e_\alpha$. In the following, we provide an expansion of $\mathcal {G}( \psi , \varphi )$ which will be useful for later proofs. For this,let $\delta _1 = \psi - \psi _\alpha$ and $\delta _2 = \varphi - \varphi _\alpha$

3.129 $$\begin{aligned} \mathcal {G}_\alpha ( \psi , \varphi ) - e_\alpha =&\mathcal {G}_\alpha ( \psi , \varphi ) - \langle \psi _\alpha |\textrm{h}_{\sqrt{\alpha }\varphi _\alpha }|\psi _\alpha \rangle - \Vert \varepsilon ^{1/2 } \varphi _\alpha \Vert _2^2 \nonumber \\ =&2 \mathrm{Re\,}\left\langle \delta _1\right| \textrm{h}_{\sqrt{\alpha }\varphi _\alpha } \left| \psi _\alpha \right\rangle +\sqrt{\alpha } \langle \psi _\alpha | V_{ \delta _2}|\psi _\alpha \rangle \nonumber \\&+ 2 \sqrt{\alpha } \mathrm{Re\,}\left\langle \varepsilon ^{1/2}\varphi _\alpha \right| \left| \varepsilon ^{1/2}\delta _2\right\rangle + \langle \delta _1|\textrm{h}_{\sqrt{\alpha }\varphi _\alpha }|\delta _1\rangle \nonumber \\&+ 2 \sqrt{\alpha } \mathrm{Re\,}\left\langle \psi _\alpha \right| V_{\delta _2} \left| \delta _1\right\rangle + \Vert \varepsilon ^{1/2}\delta _2 \Vert _2^2 + O( \sqrt{\alpha } \Vert \delta _1 \Vert _{H^1}^2 \Vert \delta _2 \Vert _2 ) \; . \end{aligned}$$

We observe that the sum of the second and third term vanish by the definition of the potential (1.3) and $\varphi _\alpha = - \sqrt{\alpha }\sigma _{\psi _\alpha }$. For the last two terms of the r.h.s., we complete the square

3.130 $$\begin{aligned} 2 \sqrt{\alpha }&\mathrm{Re\,}\left\langle \psi _\alpha \right| V_{\delta _2} \left| \delta _1\right\rangle + \Vert \varepsilon ^{1/2}\mathrm{Re\,}\delta _2 \Vert _2^2 \nonumber \\ =&\Vert \varepsilon ^{1/2} \mathrm{Re\,}\delta _2 +2 (2\pi )^{3/2} \sqrt{\alpha } \; v \varepsilon ^{-1/2} \; \widehat{ ( \mathrm{Re\,}\;\delta _1) \psi _\alpha } \Vert _2^2 \nonumber \\&- 4\alpha \langle \mathrm{Re\,}\delta _1| X_{\psi _\alpha }|\mathrm{Re\,}\delta _1\rangle \end{aligned}$$

where $X_{\psi _\alpha }$ is defined in (3.85) so that we arrive at

3.131 $$\begin{aligned} \mathcal {G}_\alpha ( \psi , \varphi ) - e_\alpha =&\langle \mathrm{Im\,}\delta _1|\textrm{h}_{\sqrt{\alpha }\varphi _\alpha } |\mathrm{Im\,}\delta _1\rangle + \Vert \varepsilon ^{1/2} \mathrm{Im\,}\delta _2 \Vert _2^2 \nonumber \\&+ 2 \mathrm{Re\,}\left\langle \delta _1\right| \textrm{h}_{\sqrt{\alpha }\varphi _\alpha } \left| \psi _\alpha \right\rangle + \langle \mathrm{Re\,}\delta _1|\left( \textrm{h}_{\sqrt{\alpha }\varphi _\alpha } - X_{\psi _\alpha } \right) | \mathrm{Re\,}\delta _1\rangle \nonumber \\&+\Vert \varepsilon ^{1/2} \mathrm{Re\,}\delta _2 +2 (2\pi )^{3/2} \sqrt{\alpha } \; v \varepsilon ^{-1/2} \; \widehat{ ( \mathrm{Re\,}\;\delta _1) \psi _{0}} \Vert _2^2 \nonumber \\&+ O(\sqrt{ \alpha } \Vert \delta _1 \Vert _2^2 \Vert \delta _2 \Vert _2 ) \; . \end{aligned}$$

The Euler-Lagrange equation of $\psi _\alpha$ together with the notation (3.85), (3.85) and the observation that by $L^2$-normalization of $\psi _\alpha$ and $\psi$

3.132 $$\begin{aligned} 1 = \Vert \psi \Vert _2^2 = \Vert \psi _\alpha + \delta _1 \Vert _2^2 = 1 + \Vert \delta _1 \Vert _2^2 + 2 \mathrm{Re\,}\left\langle \delta _1\right| \left| \psi _\alpha \right\rangle \end{aligned}$$

and therefore

3.133 $$\begin{aligned} 2 \mathrm{Re\,}\left\langle \delta _1\right| \left| \psi \right\rangle = - \Vert \delta _1 \Vert _2^2 \; \end{aligned}$$

we find with (3.85)

3.134 $$\begin{aligned} \mathcal {G}_\alpha ( \psi , \varphi ) - e_\alpha =&\langle Q_\alpha \mathrm{Im\,}\delta _1|\textrm{H}_{\alpha } |Q_\alpha \mathrm{Im\,}\delta _1\rangle + \Vert \varepsilon ^{1/2} \mathrm{Im\,}\delta _2 \Vert _2^2 \nonumber \\&+ \langle \mathrm{Re\,}\delta _1|Q_\alpha \left( \textrm{H}_{\alpha } - 4X_{\psi _\alpha } \right) Q_\alpha | \mathrm{Re\,}\delta _1\rangle \nonumber \\&+\Vert \varepsilon ^{1/2} \mathrm{Re\,}\delta _2 +2 (2\pi )^{3/2} \sqrt{\alpha } \; v \varepsilon ^{-1/2} \; \widehat{ ( \mathrm{Re\,}\;\delta _1) \psi _\alpha } \Vert _2^2 \nonumber \\&+ O(\sqrt{ \alpha } \Vert \delta _1 \Vert _2^2 \Vert \delta _2 \Vert _2 ) + O (\sqrt{\alpha } \Vert \delta _1 \Vert _2^3) + O ( \alpha \Vert \delta _1 \Vert _2^4) \; . \end{aligned}$$

##### Proof of Corollary 3.1

In order to prove (3.127) first, we remark that it suffices to consider $\psi \in H^1 ( \mathbb {R}^3)$ such that

3.135 $$\begin{aligned} \mathrm{Im\,}\left\langle e^{i \theta }\psi _\alpha ^{y}\right| \left| \psi \right\rangle = 0, \quad \text {and} \quad \mathrm{Re\,}\left\langle e^{i\theta }\psi _\alpha ^{y}\right| \left| \nabla \psi \right\rangle = 0 \end{aligned}$$

hold. In particular we assume w.l.o.g. that $\theta =0$ and $y = 0$.

Furthermore, completing the square we get

3.136 $$\begin{aligned} \mathcal {G}_\alpha ( \psi , \varphi ) -e_\alpha \ge&\mathcal {E}_\alpha ( \psi ) - e_\alpha \end{aligned}$$

and thus it suffices to consider the case $\delta _2 = \sqrt{\alpha } ( \sigma _\psi - \sigma _{\psi _\alpha })$. It follows from (3.134) and (3.135)

3.137 $$\begin{aligned} \mathcal {E}_\alpha ( \psi ) -e_\alpha \ge \langle \mathrm{Im\,}\delta _1|\textrm{H}_{\alpha } |\mathrm{Im\,}\delta _1\rangle + \langle \mathrm{Re\,}\delta _1|Q_\alpha ' \left( \textrm{H}_{\alpha } - 4 X_{\psi _\alpha } \right) Q'_\alpha | \mathrm{Re\,}\delta _1\rangle + O ( \alpha \Vert \delta _1 \Vert _2^3) \; . \end{aligned}$$

We recover back the Hessian of $\mathcal {E}_\alpha$ which by Lemma 3.3 is positive for sufficiently large $\alpha \ge \alpha _0$. Moreover, it follows from Lemma 3.3 that there exists $C_1>0$ (independent of $\alpha$) such that with $\Vert \delta _1 \Vert _2 \le \delta \alpha ^{-1/2}$ for sufficiently small $\delta >0$ by assumption, we have

3.138 $$\begin{aligned} \mathcal {E}_\alpha ( \psi )- e_\alpha \ge C_1 \sqrt{\alpha } \Vert \delta _1 \Vert _2^2 \; . \end{aligned}$$

Moreover, since $\cos ( k \cdot x) \le 1$ by Lemma 3.2 there exists a constants $\kappa _1,\kappa _2 >0$ such that

3.139 $$\begin{aligned} \textrm{h}_{\sqrt{\alpha }\varphi _\alpha } - e_\alpha \ge - \kappa _1 \Delta - \kappa _1 \sqrt{\alpha } \; . \end{aligned}$$

Interpolating between (3.138) and (3.139), there exists a constant $C_2 >0$ such that for $\alpha \ge \alpha _0$

3.140 $$\begin{aligned} \mathcal {E}_\alpha ( \psi )- e_\alpha \ge C_2 \alpha ^{1/4} \Vert \delta _1 \Vert _{H^1}^2 \; . \end{aligned}$$

By translational and rotational invariance of the energy, we conclude

3.141 $$\begin{aligned} \mathcal {E}_\alpha ( \psi )- e_\alpha \ge C_2 \alpha ^{1/4} {{\,\textrm{dist}\,}}_{H^1} \left( \Theta (\psi _\alpha ), \; \psi \right) \; . \end{aligned}$$

Second we prove (3.128): Completing the square leads to

3.142 $$\begin{aligned} \mathcal {G}_\alpha ( \psi , \varphi ) -e_\alpha =&\mathcal {E}_\alpha ( \psi ) - e_\alpha + \Vert \mathrm{Re\,}\varphi + \sqrt{\alpha } \sigma _{\psi } \Vert _{L^2_{\sqrt{\varepsilon }}}^2 + \Vert \mathrm{Im\,}\varphi \Vert _{L_{\sqrt{\varepsilon }}^2} \end{aligned}$$

so that we find from (3.138) that there exists $y \in \mathbb {R}^3$ and $\kappa _1 >0$ such that

3.143 $$\begin{aligned} \mathcal {G}_\alpha ( \psi , \varphi ) -e_\alpha \ge&\sqrt{\alpha } \kappa _1 \Vert \psi - \psi _\alpha ^y \Vert _{2}^2 + \Vert \mathrm{Re\,}\varphi + \sqrt{\alpha } \sigma _{\psi } \Vert _{L^2_{\sqrt{\varepsilon }}}^2 + \Vert \mathrm{Im\,}\varphi \Vert _{L^2_{\sqrt{\varepsilon }}}^2 \; . \end{aligned}$$

By regularity of $\varepsilon , v$, there exists $\kappa _3 >0$ such that

3.144 $$\begin{aligned} \mathcal {G}( \psi , \varphi ) -e_\alpha \ge&\kappa _3 \sqrt{\alpha } \; \Vert \sigma _{\psi } - \sigma _{\psi _\alpha ^y} \Vert _{L^2_{\sqrt{\varepsilon }}}^2+ \Vert \varphi + \sqrt{\alpha } \sigma _{\psi } \Vert _{L^2_{\sqrt{\varepsilon }}}^2 \end{aligned}$$

and we find by completing the square

3.145 $$\begin{aligned} \mathcal {G}( \psi , \varphi ) -e _\alpha \ge&\Vert ( 1 + \kappa _3/\sqrt{\alpha } )^{1/2} \left( \sigma _{\psi } - \sigma _{\psi _\alpha ^y} \right) - ( 1 +\kappa _3/ \sqrt{\alpha } )^{-1/2} \sqrt{\alpha } \left( \varphi +\sqrt{\alpha } \sigma _{\psi _\alpha ^y} \right) \Vert _{L^2_{\sqrt{\varepsilon }}}^2 \nonumber \\&+ \frac{ \kappa _3}{\sqrt{\alpha } + \kappa _3} \Vert \varphi + \sqrt{\alpha } \sigma _{\psi _\alpha ^y} \Vert _{L^2_{\sqrt{\varepsilon }}}^2 + \Vert \mathrm{Im\,}\varphi \Vert _{L^2_{\sqrt{\varepsilon }}}^2 \nonumber \\ \ge&\frac{\kappa _4}{\sqrt{\alpha }} \; \Vert \varphi + \sqrt{\alpha } \sigma _{\psi _\alpha ^y} \Vert _{L^2_{\sqrt{\varepsilon }}}^2 + \Vert \mathrm{Im\,}\varphi \Vert _{L^2_{\sqrt{\varepsilon }}}^2 \end{aligned}$$

for a constant $\kappa _4 >0$. We conclude that

3.146 $$\begin{aligned} \mathcal {G}( \psi , \varphi ) -e _\alpha \ge&\frac{\kappa _4}{\sqrt{\alpha }} {{\,\textrm{dist}\,}}_{L^2_{\sqrt{\varepsilon }} } \left( \Omega ( \varphi _\alpha ), \; \mathrm{Re\,}\varphi \right) \; . \end{aligned}$$

$$\square $$

#### Global coercivity estimates

The Hessian’s positivity shows the validity of global coercivity estimates of the energy. For this, we additionally have to assume that the ground state $\psi _\alpha$ is unique up to translations and phase, i.e. that $\mathcal {M}_{\mathcal {E}_\alpha } = \Theta ( \psi _\alpha )$. We remark that to prove the ground states uniqueness up to translations and phase by the local coercivity estimates Corollary 3.1, one needs an improved approximation of the ground state than in comparison to Proposition 2.2.

##### Corollary 3.2

Assume that the ground state $\psi _\alpha$ of $\mathcal {E}_\alpha$ is unique up to translations and rotations. There exists universal constants $\alpha _0 \ge 0$ and $C >0$ (independent of $\alpha$) such that for any $L^2$-normalized $\psi \in H^1 ( \mathbb {R}^3)$ and $\varphi \in L^2 ( \mathbb {R}^3)$ with we have for all $\alpha \ge \alpha _0$

3.147 $$\begin{aligned} \mathcal {G}_\alpha ( \psi , \varphi ) - e_\alpha&\ge C\sqrt{\alpha } {{\,\textrm{dist}\,}}_{L^2} \left( \Theta (\psi _\alpha ) , \; \psi \right) ^2 \; , \end{aligned}$$

3.148 $$\begin{aligned} \mathcal {G}_\alpha (\psi , \varphi ) - e_\alpha&\ge \frac{C}{\sqrt{\alpha }} {{\,\textrm{dist}\,}}_{L^2_{\sqrt{\varepsilon }}} \left( \Omega ( \varphi _\alpha ), \; \varphi \right) ^2 \; . \end{aligned}$$

The proof follows the arguments presented in [24, Lemma 2.6].

##### Proof

We first prove the global bound (3.147). Then the second bound (3.148) follows similarly to the proof of Corollary 3.1.

In order to prove (3.147) we remark (similarly to the proof of Corollary 3.1) that is suffices to consider $\psi \in H^1 ( \mathbb {R}^3)$ such that

3.149 $$\begin{aligned} \mathrm{Im\,}\left\langle e^{i \theta }\psi _\alpha ^{y}\right| \left| \psi \right\rangle = 0, \quad \text {and} \quad \mathrm{Re\,}\left\langle e^{i\theta }\psi _\alpha ^{y}\right| \left| \nabla \psi \right\rangle = 0 \;. \end{aligned}$$

W.l.o.g. we assume $$y=0 $$ and $$\theta =0$$. By contradiction we assume that there does not exist a universal constant $$C>0$$ such that (3.147) holds. Then there exists a sequence of functions $$\psi _n \in L^2( \mathbb {R}^3)$$ with $$\Vert \psi _n \Vert _{L^2( \mathbb {R}^3)} =1 $$ such that

3.150 $$\begin{aligned} \mathcal {E}_\alpha ( \psi _n ) \le e_\alpha + \frac{1}{n} \Vert \psi _n - \psi _\alpha \Vert _{H^1}^2 \le \frac{2}{n} \Vert \psi _n \Vert _{H^1} - C \alpha \; . \end{aligned}$$

It follows that $\mathcal {E}_\alpha ( \psi _n ) \ge \frac{1}{2} \Vert \nabla \psi _n \Vert _2^2 - C \alpha$. Therefore $\psi _n$ is uniformly bounded in $H^1$ and moreover a minimizing sequence. With similar arguments as in the proof of Lemma 3.1$\psi _n$ converges to an element of the set of minimizes $\Theta (\psi _\alpha )$ given by (3.149) through $\psi _\alpha$. This is a contradiction since Corollary 3.1 shows that locally coercivity estimates hold true. $\square$

Another consequence of the Hessian’s approximate behavior is the following property.

##### Corollary 3.3

There exists $\alpha _0$ and a constant $C>0$ (independent of $\alpha$) such that for all $\alpha \ge \alpha _0$, we have ${\textrm{H}}_\alpha - e_\alpha > C \sqrt{\alpha }$.

##### Proof

The existence of a spectral gap of ${\textrm{H}}_\alpha$ of order $\sqrt{\alpha }$ follows immediately from the global coervitiy estimates in Corollary 3.2. $\square$

### Proof of Propositions 2.1, 2.2

In this section we prove Proposition 2.1, 2.2 based on the results proven before.

#### Proof of Proposition 2.1

The proposition follows immediately from Lemma 3.1. $\square$

#### Proof of Proposition 2.2

The proposition follows from Lemma 3.2 and Corollary 3.2. $\square$

## Proof for traveling waves

In this section, we prove Proposition 2.3 on existence of subsonic traveling waves of the regularized Landau-Pekar equations.

For this, we remark that it follows from the regularized polaron’s dynamics that the traveling wave (1.5) satisfies

4.1 $$\begin{aligned} - i \textrm{v}\cdot \nabla \psi _\textrm{v}= \left( \textrm{h}_{\varphi _\textrm{v}} + e_\textrm{v}\right) \psi _\textrm{v}, \quad -\varepsilon ^{-1}\textrm{v}\cdot k \varphi _\textrm{v}= \varphi _\textrm{v}+ \sqrt{\alpha } \sigma _{\psi _\textrm{v}} \; . \end{aligned}$$

### Proof of Proposition 2.3

**Proof of** *(a)***:** First, we prove the existence of traveling waves for sufficiently small velocities. Traveling wave solutions of (1.5) are stationary points of the action functional $\mathcal {I}_\textrm{v}$ given by

4.2 $$\begin{aligned} \mathcal {J}_\textrm{v}(\psi , \varphi ) := \langle \psi |\textrm{h}_{\varphi }|\psi \rangle + \Vert \varepsilon ^{1/2} \varphi \Vert _2^2+e_\textrm{v}\Vert \psi \Vert _2^2 - \textrm{v}\cdot \left( \langle \psi |i\nabla |\psi \rangle + \langle \varphi |p |\varphi \rangle \right) \; . \end{aligned}$$

In the following we show that there exists a minimizer $(\psi _\textrm{v}, \varphi _\textrm{v})$ of $\mathcal {J}_\textrm{v}$, and thus a traveling wave solution. Since

4.3 $$\begin{aligned} \vert \textrm{v}\vert \big \vert \langle \psi |i \nabla |\psi \rangle \big \vert \le \frac{1}{2} \Vert \nabla \psi \Vert _2^2 + 2 \textrm{v}^2 \Vert \psi \Vert _2^2, \quad \vert \textrm{v}\vert \big \vert \langle \varphi |i k|\varphi \rangle \big \vert \le \vert \textrm{v}\vert \Vert \vert p \vert ^{1/2} \varphi \Vert _2^2 \end{aligned}$$

and for arbitrary $\delta >0$

4.4 $$\begin{aligned} \langle \psi |V_\varphi |\psi \rangle \le C \Vert \varepsilon ^{1/2} \varphi \Vert _2 \Vert \psi \Vert _2^2 \le \delta \Vert \varepsilon ^{1/2} \mathrm{Re\,}\varphi \Vert _2^2 + C_\delta \alpha \Vert \psi \Vert _2^4 \; , \end{aligned}$$

the action functional is bounded from below by

4.5 $$\begin{aligned} \mathcal {J}_\textrm{v}(\psi , \varphi )&\ge \frac{1}{2} \Vert \nabla \psi \Vert _2^2 + \left( 1 - \delta \right) \Vert \varepsilon ^{1/2} \varphi \Vert _2^2 - \vert \textrm{v}\vert \Vert \vert p \vert ^{1/2} \varphi \Vert _2^2 -C_\delta \alpha - C \textrm{v}^2 \end{aligned}$$

We remark that in the last step we used the $L^2$-normalization of $\psi$. By Assumption 1.2, we have $\varepsilon (k)\ge \textrm{v}_\textrm{crit}\vert k \vert$ and thus,

4.6 $$\begin{aligned} \mathcal {J}_\textrm{v}(\psi , \varphi )&\ge \frac{1}{2} \Vert \nabla \psi \Vert _2^2 + \left( \textrm{v}_\textrm{crit}( 1- \delta ) - \textrm{v}\right) \Vert \vert p \vert ^{1/2} \varphi \Vert _2^2 -C_\delta \alpha - C \textrm{v}^2 \; . \end{aligned}$$

For $\vert \textrm{v}\vert \le \textrm{v}_\textrm{crit}- \delta$, it follows that any minimizing sequence $(\psi _n,\varphi _n)_{n \in \mathbb {N}}$ of $\mathcal {I}_\textrm{v}$ is uniformly bounded in $H^1( \mathbb {R}^3) \times L^2_{\sqrt{\vert \cdot \vert }}(\mathbb {R}^3)$. Any minimizing sequence, thus, uniformly bounded and weakly converging in $H^1 ( \mathbb {R}^3) \times L_{\sqrt{\varepsilon }} ( \mathbb {R}^3)$ to a limiting functional that is possibly zero (by translational invariance of the action). With similar arguments as after Eq. (3.3), there exists a sequence $( \psi _n^{y_n}, e^{i p_n }\varphi _n)_{n \in \mathbb {N}}$ that converges strongly in $L^2(\mathbb {R}^3) \times L^2_{\sqrt{\varepsilon }}( \mathbb {R}^3)$ to a pair of non-zero limiting functions. By semi-lower continuity of the $H^1$- and the $L_{\sqrt{\varepsilon }}$-norm, and

4.7 $$\begin{aligned} \vert \langle \psi _n|V_{\varphi _n}|\psi _n\rangle - \langle \psi _\textrm{v}|V_{\varphi _\textrm{v}}|\psi _\textrm{v}\rangle \vert \le C \Vert \psi _n - \psi _\textrm{v}\Vert _2 + C \Vert \varphi _n - \varphi _\textrm{v}\Vert _{L^2_{\sqrt{\varepsilon }}} \end{aligned}$$

we conclude that the action functional $\mathcal {J}_\textrm{v}$ attains its infimum for $( \psi _\textrm{v}, \varphi _\textrm{v})$ which is a non-zero traveling wave solution (4.1).

**Proof of scaling properties:** As a preliminary step towards proving Proposition 2.3*(b)* we shall first prove that

4.8 $$\begin{aligned} \Vert x^2 \psi _\textrm{v}\Vert _2 \le C \alpha ^{-1/2} \; \end{aligned}$$

i.e. that the traveling wave satisfies similar scaling properties as the harmonic oscillator. We proceed similarly as in the proof of Lemma 3.2*(b)*. For this let $\textrm{H}_0:= - \Delta /(2\,m) + i \textrm{v}\cdot \nabla$. Then the traveling wave equation (4.1) implies

4.9 $$\begin{aligned} \left( \textrm{H}_0 + e_\textrm{v}\right) \psi _\textrm{v}= V_{\sqrt{\alpha }\varphi _\textrm{v}} \psi _\textrm{v}\; . \end{aligned}$$

Since $\textrm{H}_0 \ge - \textrm{v}^2/4$ and $e_\textrm{v}\ge - e_\alpha + \textrm{v}^2/4$, the resolvent $\left( \textrm{H}_0 + e_\textrm{v}\right) ^{-1}$ is well defined and we can write

4.10 $$\begin{aligned} \psi _\textrm{v}= \left( \textrm{H}_0 + e_\textrm{v}\right) ^{-1}V_{\sqrt{\alpha }\varphi _\textrm{v}} \psi _\textrm{v}\; . \end{aligned}$$

The resolvent’s Green’s function is given in terms of the inverse Fourier transform $\mathcal {F}^{-1}$ of

4.11 $$\begin{aligned} G_{e_\textrm{v}} (z) = \mathcal {F}^{-1} \left[ \big ( p^2 /(2m)- \textrm{v}\cdot p + e_\textrm{v}\big )^{-1} \right] (z) \end{aligned}$$

and can (by self-adjointness of $\textrm{H}_0$ and functional calculus) explicitly computed. In fact by functional calculus we have

4.12 $$\begin{aligned} \left( H_0 + e_\textrm{v}\right) ^{-1} = \int _0^\infty e^{- t ( \textrm{H}_0 + e_\textrm{v}) } \; dt = \int _0^t e^{- t e_\textrm{v}} e^{-t \left( p^2/(2m) - \textrm{v}\cdot p \right) } dt \end{aligned}$$

which leads with (4.11) to

4.13 $$\begin{aligned} G_{e_\textrm{v}} (z)&= \frac{1}{\left( 2 \pi \right) ^{3/2}} \int _{\mathbb {R}^3} \int _0^t e^{i z \cdot p} e^{-t e_\textrm{v}} e^{-t \left( p^2/(2m) - \textrm{v}\cdot p \right) } dt dp \end{aligned}$$

and we arrive with Fubini’s theorem at

4.14 $$\begin{aligned} G_{e_\textrm{v}} (z) = (2m)^{3/2}e^{i z \cdot \textrm{v}}\int _0^t e^{- t \left( e_\textrm{v}- \frac{\textrm{v}^2}{4}\right) } \frac{e^{-\frac{mz^2}{2t}}}{ t^{3/2} } dt = (2m)^{3/2} \frac{\sqrt{\pi }e^{i z \cdot \textrm{v}}}{\vert z \vert } e^{-\sqrt{4 m \left( e_\textrm{v}- \frac{\textrm{v}^2}{4}\right) } \vert z \vert } \end{aligned}$$

for $\vert z \vert \not =0$. We plug this identity into (4.10) and find that

4.15 $$\begin{aligned} \psi _\textrm{v}(x) = \int G_{e_\textrm{v}} (x-y) V_{\sqrt{\alpha } \varphi _\textrm{v}} (y) \; \psi _\textrm{v}(y) \; . \end{aligned}$$

Thus the weighted $L^2$-norm, we aim to find an upper bound for, becomes

4.16 $$\begin{aligned} \int x^{6} \vert \psi _\textrm{v}(x) \vert ^2 dx =&\int x^{6} G_{e_\textrm{v}} (x-y) V_{\sqrt{\alpha } \varphi _\textrm{v}} (y) \; \psi _\textrm{v}(y) G_{e_\textrm{v}}( x- z) V_{\sqrt{\alpha } \varphi _\textrm{v}} (z) \psi _\textrm{v}(z) dxdydz \end{aligned}$$

that we can estimate by Cauchy Schwarz with

4.17 $$\begin{aligned} \int x^{6} \vert \psi _\textrm{v}(x) \vert ^2 dx \le&\int x^{2} \; \vert G_{e_\textrm{v}} (x-y)\vert ^2 \vert V_{\sqrt{\alpha } \varphi _\textrm{v}} (y) \; \psi _\textrm{v}(y) \vert ^2 dxdy \nonumber \\ =&\pi \int \frac{ x^{6}}{ \vert x -y \vert ^2 } e^{-2 \sqrt{4m \left( e_\textrm{v}- \frac{v^2}{4}\right) } \vert x-y \vert } \vert V_{\sqrt{\alpha }\varphi _\textrm{v}} (y) \psi _\textrm{v}(y) \vert ^2 dydx \; . \end{aligned}$$

With

4.18 $$\begin{aligned} \Vert V_{\sqrt{\alpha }\varphi _{\textrm{v}}} \psi _{\textrm{v}} \Vert _2^2 \le C \alpha \Vert \varphi _\textrm{v}\Vert _{L_{\sqrt{\varepsilon }}}^2 \Vert \psi _\textrm{v}\Vert _{2}^2 \end{aligned}$$

and $\Vert \varphi _\textrm{v}\Vert _{L_{\sqrt{\varepsilon }}} \le C \sqrt{\alpha }$ from Lemma 2.1, we can use a similar splitting of the above integral as in the proof of Lemma 3.2*(b)* to then conclude by $e_\textrm{v}- \textrm{v}^2/4 \ge - e_\alpha \ge C \alpha$ with (4.8).

**Proof of** (b)**:** We observe that for $\textrm{v}= 0$ a traveling wave solution is given by $\psi _{\textrm{v}=0} = \psi _\alpha ^y, \varphi _{\textrm{v}= 0} = \varphi _\alpha ^y$ with $e_{\textrm{v}} = \mu _{\psi _\alpha ^y}$ for any $y \in \mathbb {R}$. To prove (2.15) it follows (similarly to the proof of Corollary 3.1) that it suffices to consider the decomposition

4.19 $$\begin{aligned} \psi _{\textrm{v}} = \psi _\alpha ^y + \delta _1, \quad \varphi _\textrm{v}= e^{i y \cdot }\varphi _\alpha + \delta _2, \quad \text {and} \quad e_{\textrm{v}} = \mu _{\psi _\alpha ^y} + \mu _\textrm{v}\; \end{aligned}$$

with $\left\langle \mathrm{Re\,}\delta _1\right| \left| \nabla \psi _\alpha ^y\right\rangle = 0$, $\left\langle \mathrm{Im\,}\delta _1\right| \left| \psi _\alpha ^y\right\rangle =0$. In particular it follows from Corollary 3.2 and condition (i) that

4.20 $$\begin{aligned} \Vert \delta _1 \Vert _2 \le \kappa _1 \alpha ^{-1/4}, \quad \Vert \delta _2 \Vert _{L_{\sqrt{\varepsilon }}} \le \kappa _2 \alpha ^{1/4} \quad \text {and} \quad \mu _\textrm{v}\le \kappa _3 \sqrt{\alpha } \end{aligned}$$

for sufficiently small $\kappa _1, \kappa _2, \kappa _3 >0$ (independent of $\alpha$). In the following we assume w.l.o.g. that $y=0$.

By definition of ${\textrm{H}}_\alpha$ (see (3.85)) and the decomposition (4.19), we can write the traveling wave equations (4.1) as

4.21 $$\begin{aligned} -i \textrm{v}\cdot \nabla \left( \psi _\alpha + \delta _1 \right) =&\left( V_{\sqrt{\alpha }\mathrm{Re\,}\delta _2} + \mu _{\textrm{v}} \right) \psi _\alpha + \left( { \mathrm H}_\alpha + V_{\sqrt{\alpha } \mathrm{Re\,}\delta _2} + \mu _\textrm{v}\right) \delta _1 \end{aligned}$$

4.22 $$\begin{aligned} \varepsilon ^{-1}\textrm{v}\cdot k \left( \varphi _\alpha + \delta _2 \right) =&\delta _2 + 2( 2 \pi )^{3/2} \sqrt{\alpha } v\varepsilon ^{-1} \left( \widehat{\psi _\alpha \mathrm{Re\,}\delta _1} \right) + \sqrt{\alpha } \sigma _{\delta _1} \; \end{aligned}$$

and it follows that the phase $\mu _\textrm{v}$ is given through the identity

4.23 $$\begin{aligned} \mu _\textrm{v}( 1 - \frac{1}{2}\Vert \delta _1 \Vert _2^2) = \textrm{v}\cdot \left\langle \psi _\alpha \right| \left| \nabla \mathrm{Im\,}\delta _1\right\rangle - \left\langle \psi _\alpha \right| V_{\sqrt{\alpha } \mathrm{Re\,}\delta _2} \left| \psi _\alpha \right\rangle - \left\langle \psi _\alpha \right| V_{\sqrt{\alpha } \mathrm{Re\,}\delta _2} \left| \mathrm{Re\,}\delta _1\right\rangle \; . \end{aligned}$$

Plugging this identity back into (4.21), we get

4.24 $$\begin{aligned} \left( {\textrm{H}}_\alpha - A \right) \delta _1 =&- Q_{\textrm{v}} \left( V_{\sqrt{\alpha } \mathrm{Re\,}\delta _2} \psi _\textrm{v}- \textrm{v}\cdot \nabla \psi _\textrm{v}\right) \end{aligned}$$

where we introduced the notation $Q_\textrm{v}= 1- \left| \psi _\textrm{v}\right\rangle \left\langle \psi _\textrm{v}\right|$ and the operator

4.25 $$\begin{aligned} A =&\frac{\Vert \delta _1 \Vert _2^2}{2 - \Vert \delta _1 \Vert _2^2} \left( \textrm{v}\cdot \left\langle \psi _\alpha \right| \left| \nabla \mathrm{Im\,}\delta _1\right\rangle - \left\langle \psi _\alpha \right| V_{\sqrt{\alpha } \mathrm{Re\,}\delta _2} \left| \mathrm{Re\,}\delta _1\right\rangle - \left\langle \psi _\alpha \right| V_{\sqrt{\alpha } \mathrm{Re\,}\delta _2} \left| \psi _\alpha \right\rangle \right) \; . \end{aligned}$$

With the decomposition’s properties (4.20) we find that

4.26 $$\begin{aligned} \Vert A \Vert \le C \Vert \delta _1 \Vert _2^2 \left( \sqrt{\alpha } \Vert \delta _2 \Vert _2 + \sqrt{\alpha }\textrm{v}\right) \le C ( \alpha ^{1/4} + \textrm{v}) \; . \end{aligned}$$

Thus by Corollary 3.3 there exists $\kappa _4 >0$ such that by assumption $Q_{\textrm{v}}({\textrm{H}}_\alpha + A )Q_{\textrm{v}} \ge \kappa _4 \sqrt{\alpha }$, i.e. we can write

4.27 $$\begin{aligned} \delta _1 = \frac{Q_{\textrm{v}}}{{\textrm{H}}_\alpha - A} \left( V_{\sqrt{\alpha } \mathrm{Re\,}\delta _2} \psi _\textrm{v}- \textrm{v}\cdot \nabla \psi _\textrm{v}\right) \;. \end{aligned}$$

The second term of the r.h.s. leads with Proposition 3.2 to the desired bound. For the first term we observe that by definition of the potential and radialilty of *v*

4.28 $$\begin{aligned} Q_{\textrm{v}} V_{\sqrt{\alpha } \delta _2} \psi _\textrm{v}=&Q_{\textrm{v}} V_{\sqrt{\alpha } \delta _2^s} \psi _\textrm{v}\end{aligned}$$

where $\delta _2^s$ denotes the symmetric part of $\delta _2$, i.e. $\delta _2^s(k) = \delta _2^s( -k )$. We observe that splitting $\delta _2$ into its symmetric $\delta _2^{s} (k) = \delta _2^{s} (-k)$ and anti-symmetric $\delta _2^{a} (k) = - \delta _2^{a} (-k)$ we have from the traveling wave Eq. (4.22)

4.29 $$\begin{aligned} \delta _2^{s} = \frac{\varepsilon ^{-2} ( k \cdot \textrm{v})^2}{1-\varepsilon ^{-2} ( k \cdot \textrm{v})^2} \varphi _\alpha - \frac{\sqrt{\alpha }}{1-\varepsilon ^{-2} ( k \cdot \textrm{v})^2} \left( 2 (2 \pi )^{3/2} v\varepsilon ^{-1} \widehat{\mathrm{Re\,}\delta _1 \psi _\alpha } + \; \sigma _{\delta _1} \right) \; . \end{aligned}$$

Here we used that $\varepsilon ^{-2} ( k \cdot \textrm{v})^2 \le \textrm{v}^2/ \textrm{v}_\textrm{crit}^2 <1$ by Assumption 1.2. Hence

4.30 $$\begin{aligned} Q_{\textrm{v}} V_{\sqrt{\alpha } \mathrm{Re\,}\delta _2} \psi _\textrm{v}=&2 \alpha Q_\textrm{v}\int e^{ik \cdot } \frac{\varepsilon ^{-2} ( k \cdot \textrm{v})^2}{1-\varepsilon ^{-2} ( k \cdot \textrm{v})^2} \varphi _\alpha (k) \; dk \; \; \psi _\textrm{v}\; \nonumber \\&- \alpha Q_\textrm{v}\int e^{ik \cdot } \frac{ \widehat{h} (k) }{1-\varepsilon ^{-2} ( k \cdot \textrm{v})^2} \left( 2 \left( \widehat{\mathrm{Re\,}\delta _1 \psi _\alpha }\right) (k) + \; \widehat{\varrho }_{\delta _1} (k) \right) dk \; \; \psi _\textrm{v}\; . \end{aligned}$$

We observe that due to the projection $Q_\textrm{v}$ the first term of the Taylor expansion of $\cos ( k \cdot )$ vanishes. Thus with $\varepsilon ^{-2} ( k \cdot \textrm{v})^2 \le \textrm{v}^2/ \textrm{v}_\textrm{crit}^2 <1$ and $\left\langle \mathrm{Re\,}\psi _\alpha \right| \left| \psi _\alpha \right\rangle = - \Vert \delta _1 \Vert _2^2$ we arrive at

4.31 $$\begin{aligned} \Vert V_{\sqrt{\alpha } \delta _2^s} \delta _1 \Vert _2 \le&C\textrm{v}^2 \alpha \left( \Vert x^2 \psi _\textrm{v}\Vert _2 + \Vert x \psi _\alpha \Vert _2 \Vert x \psi _\textrm{v}\Vert _2 \right) \nonumber \\&+ C \alpha \left( \Vert x^2 \psi _\textrm{v}\Vert _2 \Vert \delta _1 \Vert _2^2 + \Vert \delta _1 \Vert _2 \Vert x \psi _\alpha \Vert _2 \Vert x \psi _\textrm{v}\Vert _2 + \Vert \delta _1 \Vert _2 \Vert x \delta _1 \Vert _2 \Vert x \psi _\textrm{v}\Vert _2\right) \; . \end{aligned}$$

From the scaling properties of the traveling wave (4.8) and the ground state (see Corollary 3.3) we conclude

4.32 $$\begin{aligned} \Vert V_{\sqrt{\alpha } \delta _2^s} \delta _1 \Vert _2 \le C \sqrt{\alpha } \left( \textrm{v}^2 + \kappa _1\alpha ^{-1/4} \Vert \delta _1 \Vert _2 \right) \end{aligned}$$

yielding for $$\vert \textrm{v}\vert \le 1$$

4.33 $$\begin{aligned} \left( 1- \kappa _1 \alpha ^{-1/4} \right) \Vert \delta _1 \Vert _2 \le C \vert \textrm{v}\vert \end{aligned}$$

and (2.18) follows from (4.22) resp. (4.23)

4.34 $$\begin{aligned} \Vert \delta _2 \Vert \le C \sqrt{\alpha } \vert \textrm{v}\vert \quad \text {resp.} \quad \mu _{\textrm{v}} \le \alpha ^{3/4} \textrm{v}^2 \; . \end{aligned}$$

$$\square $$

## Proofs for definitions effective mass

In this section, we prove Theorems 2 and 3 on the definition of the effective mass.

We remark that the proofs presented in this Section follow ideas from [11] where the non-regularized Landau-Pekar equations have been considered. For the non-regularized Landau-Pekar equations traveling waves are conjectured to not exists. However assuming their existence an energy expansion in the vein of the proof of Theorem 2 was sketched. Furthermore a different approach for a definition of the mass through an energy-velocity expansion was presented. The proof of Theorem 3 given below uses ideas presented there.

### Effective mass through traveling waves

We consider the definition of the effective mass through subsonic traveling waves first (whose existence follow from Proposition 2.3).

#### Proof of Theorem 2

Let $\alpha \ge \alpha _0$ large enough and $\vert \textrm{v}\vert < \textrm{v}_\textrm{crit}$. Then, by Proposition 2.3 and 2.1, there exists $y,z \in \mathbb {R}^3$ and $\theta \in (0, 2 \pi ]$ such that

5.1 $$\begin{aligned} \Vert e^{i \theta } \psi _\alpha ^y - \psi _\textrm{v}\Vert _2 \le C \textrm{v}, \quad \Vert e^{i z \cdot } \varphi _\alpha - \varphi _\textrm{v}\Vert _{L_{\sqrt{\varepsilon }}^2} \le C \sqrt{\alpha } \vert \textrm{v}\vert \; \end{aligned}$$

with $\left\langle \mathrm{Im\,}\psi _\textrm{v}\right| \left| e^{i \theta } \psi _\alpha \right\rangle = 0$, $\left\langle \mathrm{Re\,}\nabla \psi _\textrm{v}\right| \left| e^{i \theta }\psi _\alpha ^y\right\rangle = 0$ and $\left\langle e^{i z \cdot }\varphi _\alpha \right| \left| \nabla \varphi _\textrm{v}\right\rangle =0$ and $\psi _\alpha$ is uniquely given (up to translations and changes of phase). W.l.o.g. we assume in the following $y=0, \theta =0$. Then, (3.145) shows (with similar arguments as used in [11]) that we it suffices to consider $z =0$, too. Thus, we decompose the traveling wave as

5.2 $$\begin{aligned} \left( \psi _\textrm{v}, \varphi _\textrm{v}\right) = \left( \psi _\alpha + \textrm{v}\xi _\textrm{v}, \; \varphi _\alpha + \textrm{v}\eta _\textrm{v}\right) \end{aligned}$$

with $\Vert \xi _\textrm{v}\Vert _2 \le C$ and $\Vert \eta _\textrm{v}\Vert _{L^2_{\sqrt{\varepsilon }}} \le C \sqrt{\alpha }$. Note that Proposition 2.3 moreover shows that $e_\textrm{v}= \mu _{\psi _\alpha } + O( \alpha ^{3/4}\textrm{v}^2)$ and the linearisation of the traveling wave Eq. (4.1) read

5.3 $$\begin{aligned} \begin{pmatrix} \nabla \psi _\alpha \\ k \varphi _\alpha \end{pmatrix} = \begin{pmatrix} {\textrm{H}}_\alpha &  (2\pi )^{3/2} \sqrt{\alpha } \psi _\alpha \int dk \; v(k) e^{ik \cdot } \\ (2\pi )^{3/2} \sqrt{\alpha } \int dk \; v(k) e^{ik\cdot } \psi _\alpha &  \varepsilon \end{pmatrix} \begin{pmatrix} \xi _\textrm{v}\\ \eta _\textrm{v}\end{pmatrix} \; . \end{aligned}$$

In particular, it follows

5.4 $$\begin{aligned}&\textrm{H}_{\alpha } \mathrm{Im\,}\xi _\textrm{v}= \nabla \psi _\alpha \end{aligned}$$

5.5 $$\begin{aligned}&\varepsilon \mathrm{Im\,}\eta _\textrm{v}= k \varphi _\alpha \end{aligned}$$

5.6 $$\begin{aligned}&\textrm{H}_{\varphi _\alpha } \mathrm{Re\,}\xi _\textrm{v}+ \sqrt{\alpha } \psi _\alpha V_{\mathrm{Re\,}\eta _\textrm{v}} = 0 \end{aligned}$$

5.7 $$\begin{aligned}&\sqrt{\alpha } V_{\psi _\alpha \mathrm{Re\,}\xi _\textrm{v}} + \varepsilon \mathrm{Re\,}\eta _\textrm{v}= 0 \; . \end{aligned}$$

Combining (5.7) and (5.6), we find

5.8 $$\begin{aligned} \left( \textrm{H}_{\alpha } - 4 X_{\psi _\alpha } \right) \mathrm{Re\,}\xi _v = 0 \;. \end{aligned}$$

As $\textrm{H}_{\alpha }$ is invertible on the span of $\partial _1 \psi _\alpha$, we find from (3.131) with (5.4) and (5.5) for all $\vert \textrm{v}\vert \le C \alpha ^{-1}$

5.9 $$\begin{aligned} \mathcal {G}( \psi _\textrm{v}, \varphi _{\textrm{v}} ) = e_\alpha + \left( m +\frac{ 2 (2\pi )^3 \alpha }{3} \Vert k v \varepsilon ^{-3/2} \; \widehat{\varrho }_{\psi _\alpha } \Vert _2^2 \right) \frac{\textrm{v}^2}{2} + O(\alpha \vert \textrm{v}\vert ^3) \;. \end{aligned}$$

$$\square $$

### Effective mass through energy-momentum expansion

Here, we consider the definition of the effective mass through the energy-momentum expansion explained in Sect. 5.

#### Proof of Theorem 3

We first pick a trial state to show an upper bound on $\inf _{\mathcal {I}_\textrm{p}}\mathcal {G}(\psi , \varphi )$ which we use later for the lower bound. For this, we choose $\alpha _0 >0$ sufficiently large, such that by Proposition 2.1 (b), there exists a unique (up to translations and phases) pair of minimizers $(\psi _\alpha , \varphi _\alpha )$ of $\mathcal {G}_\alpha$.

**Proof of the upper bound of** *(a)***:** It is easy to check that the trial states

5.10 $$\begin{aligned} \psi _0 =&( 1- \mu _\textrm{p}) \lambda _p^{-1} \psi _{\alpha } +i \lambda _p \cdot \; \textrm{H}_{\alpha }^{-1} \nabla \psi _\alpha , \quad \varphi _0 (k) = \varphi _\alpha (k) + i (1- \mu _\textrm{p}) \lambda _p \cdot k \; (\varepsilon (k))^{-1} \varphi _\alpha (k) \end{aligned}$$

with the choice

5.11 $$\begin{aligned} ( 1- \mu _\textrm{p}) \lambda _\textrm{p}= \frac{\textrm{p}}{ m + \frac{ 2 (2\pi )^3 \alpha }{3} \Vert k v \varepsilon ^{-3/2} \; \widehat{\varrho }_{\psi _\alpha } \Vert _2^2} \end{aligned}$$

and $(1-\mu _\textrm{p})^2 + \lambda _\textrm{p}^2 =1$ (i.e. $\mu _p = O( \alpha ^{-2}\textrm{p}^2)$) satisfy constraint (1.15) (using that $\textrm{H}_{\alpha }^{-1} \nabla \psi _\alpha = m x \psi _\alpha$) and that for large $\alpha \ge \alpha _0$

5.12 $$\begin{aligned} \Vert \psi _\alpha - \psi _0 \Vert _2 \le C \alpha ^{-1} \textrm{p}\; \quad \text {and} \quad \Vert \varphi _\alpha - \varphi _0 \Vert _2 \le C \alpha ^{-1/2} \textrm{p}\; . \end{aligned}$$

With these observations, we plug the trail states into the expansion of $\mathcal {G}_\alpha$ in (3.134) and find

5.13 $$\begin{aligned} \mathcal {G}_\alpha ( \psi _0, \varphi _0) - e_\alpha \le&( 1- \mu _p)^2 \lambda _\textrm{p}^2 \left( \langle \nabla \psi _\alpha |\textrm{H}_{\varphi _\alpha }^{-1}| \nabla \psi _\alpha \rangle +\frac{1}{3} \Vert \varepsilon ^{-1/2} k \varphi _\alpha \Vert _2^2\right) + O(\alpha ^{-2} \textrm{p}^2) \nonumber \\ =&\frac{ ( 1- \mu _p)^2\lambda _\textrm{p}^2 }{2} \left( m + \frac{ 2 (2\pi )^3 \alpha }{3} \Vert k v \varepsilon ^{-3/2} \; \widehat{\varrho }_{\psi _\alpha } \Vert _2^2\right) + O(\alpha ^{-2} \textrm{p}^2) \nonumber \\ =&\left( m +\frac{ 2 (2\pi )^3 \alpha }{3} \Vert k v \varepsilon ^{-3/2} \; \widehat{\varrho }_{\psi _\alpha } \Vert _2^2\right) ^{-1} \frac{\textrm{p}^2}{2} + O( \alpha ^{-2} \textrm{p}^2) \; . \end{aligned}$$

**Proof of the lower bound of** *(a)***:** For $\textrm{p}\le \alpha ^{1/4}$, it follows from the upper bound and Corollary 3.2 that for any element $( \psi , \varphi ) \in \mathcal {I}_\textrm{p}$, there exists $y \in \mathbb {R}^3$ and $\theta , \omega \in (0, 2 \pi ]$ such that

5.14 $$\begin{aligned} \alpha ^{-1/4} \Vert e^{i \theta } \psi _\alpha ^y - \psi \Vert _2 = O( \alpha ^{-1/2} \textrm{p}) = \alpha ^{1/4} \Vert e^{i \omega }\varphi _\alpha - \varphi \Vert _{L^2_{\sqrt{\varepsilon }}} \; . \end{aligned}$$

W.l.o.g. we assume $y=0, \theta =0$ and, with (3.145) we consider furthermore $\omega =0$. It follows from the expansion (3.134) of the energy $\mathcal {G}_\alpha$

5.15 $$\begin{aligned} \mathcal {G}_\alpha ( \psi , \varphi ) - e_\alpha \ge&\langle \mathrm{Im\,}\delta _1|\mathrm{H_{osc}}|\mathrm{Im\,}\delta _1\rangle + \Vert \varepsilon ^{1/2} \mathrm{Im\,}\varphi \Vert _2^2 + O( \alpha ^{-5/4} \textrm{p}^3 ) \; . \end{aligned}$$

Completing the square, we find with (5.11)

5.16 $$\begin{aligned} \mathcal {G}_\alpha ( \psi , \varphi ) - e_\alpha \ge&\langle \mathrm{Im\,}\delta _1 - \lambda _\textrm{p}\textrm{H}_{\varphi _\alpha }^{-1} \nabla \psi _\alpha |\textrm{H}_{\varphi _\alpha }|\mathrm{Im\,}\delta _1 - \lambda _\textrm{p}\textrm{H}_{\varphi _\alpha }^{-1} \nabla \psi _\alpha \rangle \nonumber \\&+ \langle \mathrm{Im\,}\varphi - \lambda _\textrm{p}\varepsilon ^{-1}k \varphi _\alpha |\varepsilon | \mathrm{Im\,}\varphi - \lambda _\textrm{p}\varepsilon ^{-1}k \varphi _\alpha \rangle \nonumber \\&+ 2 \lambda _\textrm{p}\left( {\langle \nabla \psi _\alpha \rangle }{\mathrm{Im\,}\delta _1} + {\langle k \varphi _\alpha \rangle }{\varphi } \right) \nonumber \\&- \lambda _\textrm{p}^2 \langle \nabla \psi _\alpha |\mathrm{H_{osc}}^{-1}|\nabla \psi _\alpha \rangle - \lambda _\textrm{p}^2 \langle k \varphi _\alpha |\varepsilon ^{-1} | k \varphi _\alpha \rangle \nonumber \\&+ O( \alpha ^{-5/4} \textrm{p}^3 ) \; . \end{aligned}$$

For the lower bound, we can neglect the first two lines and obtain

5.17 $$\begin{aligned} \mathcal {G}( \psi , \varphi ) - e_\alpha \ge&2 \lambda _\textrm{p}( 1- \mu _p) \left( {\langle \nabla \psi _\alpha \rangle }{\mathrm{Im\,}\delta _1} + {\langle k \varphi _\alpha \rangle }{ \mathrm{Im\,}\varphi } \right) \nonumber \\&- \lambda _\textrm{p}^2( 1- \mu _p)^2 \langle \nabla \psi _\alpha |\mathrm{H_{osc}}^{-1}|\nabla \psi _\alpha \rangle - \lambda _\textrm{p}^2 \langle k \varphi _\alpha |\varepsilon ^{-1} | k \varphi _\alpha \rangle \nonumber \\&+ O( \alpha ^{-5/4} \textrm{p}^3 ) \; . \end{aligned}$$

For the first line, we use the constraint (ii)$_{\textrm{p}}$, (5.14) together with Assumption 1.2 and the trivial bound $\Vert \nabla \psi _0 \Vert \le C \sqrt{\alpha }$.The second line, we compute explicitly and obtain

5.18 $$\begin{aligned} \mathcal {G}( \psi , \varphi ) - e_\alpha \ge&\left( m +\frac{ 2 (2\pi )^3 \alpha }{3} \Vert k v \varepsilon ^{-3/2} \; \widehat{\varrho }_{\psi _\alpha } \Vert _2^2\right) ^{-1} \frac{\textrm{p}^2}{2} + O( \alpha ^{-5/4} \textrm{p}^3 ) \; . \end{aligned}$$

Combining now the upper (5.18) and the lower bound (5.13), we arrive at Theorem 3.

**Proof of** (b)**:** The corresponding Lagrange functional to the minimization problem is given by

5.19 $$\begin{aligned} \mathcal {L}_\textrm{p}( \psi , \varphi , \lambda , \mu ) := \mathcal {G}_\alpha ( \psi , \varphi ) - \lambda \left( \langle \psi |i \nabla |\psi \rangle + \langle \varphi |ik| \varphi \rangle - \textrm{p}\right) - \mu \left( \left\langle \psi \right| \left| \psi \right\rangle \right) \; . \end{aligned}$$

Thus any minimizer satisfies the traveling wave equations with velocity $\lambda$, i.e.

5.20 $$\begin{aligned} - i \lambda \partial _1 \psi _\textrm{p}= \left( h_{\varphi _\textrm{p}} - \mu \right) \psi _\textrm{p}, \quad \lambda k_1 \varphi _\textrm{p}= \varepsilon \varphi _\textrm{p}+ ( 2 \pi ) ^{3/2} \sqrt{\alpha } v \widehat{\varrho }_{\psi _\textrm{p}} \; . \end{aligned}$$

By definition of the set $\mathcal {I}_\textrm{p}$ in (1.16), Proposition 2.3 shows that we can decompose

5.21 $$\begin{aligned} \psi _\textrm{p}= \psi _\alpha + \lambda \delta _1, \quad \varphi _\textrm{p}= \varphi _\alpha + \lambda \delta _2 \end{aligned}$$

with $\Vert \delta _1 \Vert _2 \le C \alpha ^{-1/4}$ and $\Vert \varepsilon ^{1/2} \delta _2 \Vert _2 \le C \alpha ^{1/4}$. The coercivity estimates from Corollary 3.2 together with the upper bound of part *(a)* then show

5.22 $$\begin{aligned} \lambda \Vert \delta _1 \Vert _2 \le C \alpha ^{-3/4}\textrm{p}, \quad \text {and} \quad \lambda \Vert \varepsilon ^{1/2} \delta _2 \Vert _2 \le C \alpha ^{-1/4} \textrm{p}\;. \end{aligned}$$

Together with the traveling waves equation it follows from constraint (ii)$_\textrm{p}$ for all $\textrm{p}\le 1$

5.23 $$\begin{aligned} \textrm{p}= \lambda m_\textrm{eff} + O( \alpha ^{-1} \textrm{p}^2) = \lambda m_\textrm{eff} + O (\alpha ^{-1}\textrm{p}^2) \; . \end{aligned}$$

Furthermore from the first part of the theorem and Theorem 2, we conclude

5.24 $$\begin{aligned} \mathcal {G}_\alpha ( \psi _{\textrm{v}'}, \varphi _{\textrm{v}'} ) = E_\textrm{p}+ O( \alpha ^{-2} \textrm{p}^3) \; . \end{aligned}$$

where $( \psi _{\textrm{v}'}, \varphi _{\textrm{v}'} )$ denotes a traveling wave with velocity $\textrm{v}' = m_\textrm{eff}^{-1} \textrm{p}+ O( \alpha ^{-1} \textrm{p}^2)$. $\square$

## Data Availability

Data sharing not applicable to this article as no datasets were generated or analyzed during the current study.

## References

[1] Fröhlich, H.: Theory of electrical breakdown in ionic crystals. Proc. R. Soc. Lond. A **160**(901), 230–241 (1937) 10.1098/rspa.1937.0106

[2] Landau, L.: Über die Bewegung der Elektronen im Kristallgitter. Phys. Z. Sowjetunion **3**, 664 (1933)

[3] Pekar, S.: Zh. Eksp. Teor. Fiz. **16**, 335 (1946); J. Phys. USSR. **10**, 341 (1946)

[4] Landau, L., Pekar, S.: Effective mass of a Polaron. J. Exp. Theor. Phys. **18**, 419–423 (1948)

[5] Frank, R., Zhou, G.: Derivation of an effective evolution equation for a strongly coupled polaron. Anal. PDE **10**, 379–422 (2017) 10.2140/apde.2017.10.379

[6] Feliciangeli, D., Seiringer, R.: The strongly coupled Polaron on the torus: quantum corrections to the Pekar asymptotics. Arch. Ration. Mech. Anal. **242**, 1835–1906 (2021) 10.1007/s00205-021-01715-7

[7] Griesemer, M.: On the dynamics of Polarons in the strong-coupling limit. Reviews in Mathematical Physics **29**(10), 1750030 (2017) 10.1142/S0129055X17500301

[8] Leopold, N., Mitrouskas, D., Rademacher, S., Schlein, B., Seiringer, R.: Landau-Pekar equations and quantum fluctuations for the dynamics of a strongly coupled polaron. Pure Appl. Anal. **3**(4), 653–676 (2021) 10.2140/paa.2021.3.653

[9] Leopold, N., Rademacher, S., Schlein, B., Seiringer, R.: The Landau-Pekar equations: adiabatic theorem and accuracy. Anal. & PDE **14**, 2079–2100 (2021) 10.2140/apde.2021.14.2079

[10] Mitrouskas, D.: A note on the Fröhlich dynamics in the strong coupling limit. Lett. Math. Phys. **111**(2), 45 (2021) 10.1007/s11005-021-01380-7

[11] Feliciangeli, D., Rademacher, S., Seiringer, R.: The effective mass problem for the Landau-Pekar equations. J. Phys. A: Math. Theor. **55**, 015201 (2022) 10.1088/1751-8121/ac3947

[12] Brooks, M., Seiringer, R.: The Fröhlich Polaron at strong coupling–part II: energy-momentum relation and effective mass. Preprint: arXiv:2211.03353

[13] Lieb, E., Seiringer, R.: Divergence of the effective mass of a Polaron in the strong coupling limit. J. Stat. Phys. **180**, 23–33 (2020) 32801392 10.1007/s10955-019-02322-3 PMC7403139

[14] Mitrouskas, D., Myśliwy, K., Seiringer, R.: Optimal parabolic upper bound for the energy-momentum relation of a strongly coupled polaron, Preprint: arXiv:2203.02454 (2022)

[15] Bazaes, R., Mukherjee, C., Varadhan, S.R.S.: Effective mass of the Fröhlich Polaron and the Landau-Pekar-Spohn conjecture. Preprint: arXiv:2307.13058

[16] Beetz, V., Polzer, S.: Effective mass of the Polaron: a lower bound. Commun. Math. Phys. **399**, 173–188 (2023) 10.1007/s00220-022-04553-0

[17] Spohn, H.: Effective mass of the Polaron: a functional integral approach. Ann. Phys. **175**, 278–318 (1987) 10.1016/0003-4916(87)90211-9

[18] Myśliwy, K., Seiringer, R.: Polaron models with regular interactions at strong coupling. J. Stat. Phys. **186**(1), 5 (2022) 10.1007/s10955-021-02851-w

[19] Béthuel, F., Gravejat, P., Saut, J.-C.: Existence and properties of travelling waves for the Gross-Pitaevskii equation, Stationary and time dependent Gross-Pitaevskii equations. Contemp. Math. **473,** Amer. Math. Soc. Providence, RI, pp. 55–103 (2008)

[20] Fröhlich, J., Jonsson, B.L.G., Lenzmann, E.: Boson stars as solitary waves. Commun. Math. Phys. **274**, 1–30 (2007) 10.1007/s00220-007-0272-9

[21] Lieb, E.H.: Existence and uniqueness of the minimizing solution of Choquard’s nonlinear equation. Stud. Appl. Math. **57**, 93–105 (1977) 10.1002/sapm197757293

[22] Lieb, E.H.: On the lowest eigenvalue of the Laplacian for the intersection of two domains. Invent. Math. **74**, 441–48 (1983) 10.1007/BF01394245

[23] Lieb, E.H., Loss, M.: Analysis. American Mathematical Society, Providence (2001)

[24] Feliciangeli, D., Rademacher, S., Seiringer, R.: Persistence of the spectral gap for the Landau-Pekar equations. Lett. Math. Phys. **111**, 1–19 (2021) 10.1007/s11005-020-01350-5

[25] Donsker, M., Varadhan, S.: Asymptotics for the Polaron. Comm. Pure Appl. Math. **36**, 505–528 (1983) 10.1002/cpa.3160360408

[26] Frank, R., Schlein, B.: Dynamics of a strongly coupled polaron. Lett. Math. Phys. **104**, 911–929 (2014) 10.1007/s11005-014-0700-7

[27] Frank, R., Seiringer, R.: Quantum corrections to the Pekar asymptotics of a strongly coupled polaron. Commun. Pure Appl. Math. **74**, 544–588 (2021) 10.1002/cpa.21944

[28] Lieb, E., Thomas, L.: Exact ground state energy of the strong-coupling Polaron. Commun. Math. Phys. **183**, 519 (1997) 10.1007/s002200050040

[29] Simon, B.: Semiclassical analysis of low lying eigenvalues. I. Non-degenerate minima : asymptotic expansions. Annales de l’I. H. P., section A, tome. **38**(3), 295–308 (1983)

